# Applications and Advances of Liquid-State ^13^C–^13^C 2D INADEQUATE NMR in Structural Elucidation: From Small Molecules to Polymers

**DOI:** 10.3390/molecules31172974

**Published:** 2026-08-25

**Authors:** Fuyue Tian, Xuelei Duan, Xiaojie Ji, Youlin Xia, Aitor Moreno, Shan Ye, Yu Zhou, Yifei Wang, Congyun Liu, Linge Ma, Shuai Shao, Rongjuan Cong, Zhe Zhou

**Affiliations:** 1 National Institute of Clean-and-Low-Carbon Energy, Beijing 102209, China; 2 Department of Structural Biology, St. Jude Children’s Research Hospital, Memphis, TN 38105, USA; 3 Bruker Switzerland AG, CH-8117 Fällanden, Switzerland

**Keywords:** liquid-state ^13^C–^13^C 2D INADEQUATE, carbon–carbon connectivity, carbon–carbon scalar coupling, small molecules, polymers, cryoprobe, artificial intelligence

## Abstract

Liquid-state ^13^C–^13^C 2D INADEQUATE NMR spectroscopy provides unparalleled direct carbon–carbon connectivity mapping via scalar couplings, delivering unambiguous structural evidence that conventional HSQC and HMBC methods cannot, particularly for quaternary carbons, fully substituted aromatics, and overlapping polymer resonances. Despite sensitivity challenges arising from low ^13^C natural abundance (~1.1%), this review examines the technique’s evolution from its 1981 introduction to contemporary innovations, compiling over 100 application entries across natural products, synthetic molecules, mixtures, fullerenes, metabolomics, oligomers and polymers. Significant methodological advancements—including cryogenic probe technology (up to 5.5-fold sensitivity gain), J-compensated sequences, composite refocusing (INADEQUATE CR), hybrid INEPT-INADEQUATE approaches, adiabatic pulses, and Overhauser DNP-INADEQUATE—are critically assessed alongside computerized analysis algorithms (CCBond, FRED) and network-based metabolomics tools (INETA, PyINETA). Practical guidance covering optimal J_CC_ coupling selection, relaxation management, and a decision tree for experiment selection is consolidated. Looking forward, the convergence of artificial intelligence, non-uniform sampling, and integration with density functional theory promises to transform 2D INADEQUATE from a specialist technique of last resort into a routinely accessible tool for structural elucidation.

## 1. Introduction

Nuclear Magnetic Resonance (NMR) spectroscopy stands as one of the most powerful analytical techniques for determining molecular structures in solutions. Among the various NMR methodologies, the ability to directly map carbon–carbon (C-C) connectivities represents a fundamental challenge that has long captivated spectroscopists. Conventional heteronuclear correlation experiments such as Heteronuclear Single Quantum Coherence (HSQC) and Heteronuclear Multiple Bond Correlation (HMBC) provide valuable information about carbon–proton (C-H) connectivities but often fall short when it comes to establishing direct C-C bonds, particularly in proton-deficient molecules or complex natural products with quaternary centers. In such scenarios, the ^13^C–^13^C Two-Dimensional Incredible Natural Abundance Double Quantum Transfer Experiment (2D INADEQUATE) technique emerges as an indispensable tool, offering unambiguous determination of C-C connectivity networks through the direct observation of ^13^C–^13^C scalar couplings.

The 2D INADEQUATE experiment was first introduced in 1981 by Bax et al. [1], who demonstrated its utility for tracing the carbon skeleton of 5α-androstane by detecting C-C connectivities through double-quantum coherence (DQC). Their pioneering work established that this method could identify directly bonded carbon-13 pairs by measuring their double-quantum (DQ) frequencies, which correspond to the algebraic sum of their chemical shifts. Their experiment successfully resolved 37 out of 44 possible DQ frequencies in natural-abundance samples, providing definitive evidence for connectivities. In the same year, the method was applied to sucrose by Bax et al. [2]. This breakthrough represented a paradigm shift in structural elucidation, offering a direct pathway to map molecular carbon frameworks that had previously required indirect inference from proton-coupled experiments.

At the heart of the 2D INADEQUATE technique lies a specific pulse sequence that forms the foundation for all its variants. The standard pulse sequence for 2D INADEQUATE consists of four radiofrequency pulses on the ^13^C channel: 90-τ-180-τ-90-t_1_-90-AQ (τ: Echo delay time; AQ: Acquisition Time). This sequence operates through a carefully orchestrated manipulation of nuclear spin coherences to selectively detect DQ transitions between coupled carbon pairs. The initial 90° pulse excites ^13^C magnetization into the transverse plane, generating coherences that evolve under the influence of both chemical shifts and ^13^C–^13^C homonuclear couplings. The subsequent 180° refocusing pulse serves to reverse chemical-shift evolution while allowing J-coupling interactions to persist. The second 90° pulse creates DQC for the two coupled carbon spins, while efficient DQC generation requires that the τ be set to 1/(4J_CC_), where J_CC_ denotes the ^13^C–^13^C homonuclear coupling constant. In practice, an average J value (typically 45–65 Hz for one-bond couplings) is used to accommodate the distribution of coupling strengths across different C-C bonds. During the evolution delay time (t_1_), the DQC evolves at the sum frequency of the two coupled spins (ω_DQ_ = ω_1_ + ω_2_), effectively encoding the connectivity information in the indirect (F_1_) dimension of the 2D spectrum. The final 90° read pulse then converts this DQC back into observable antiphase single-quantum coherence (AP-SQC), which is detected during the AQ. Because of the AP-SQC, the two peaks of a ^13^C–^13^C doublet separated by a J_CC_ show positive and negative intensities, respectively. This elegant sequence effectively isolates the DQC pathway while suppressing unwanted single-quantum (SQ) signals, allowing for the clear identification of scalar-coupled carbon pairs in the resulting 2D spectrum. The cross-peaks along the F_1_ dimension in the 2D INADEQUATE spectrum correspond to a pair of directly bonded carbons.

**Figure 1:**
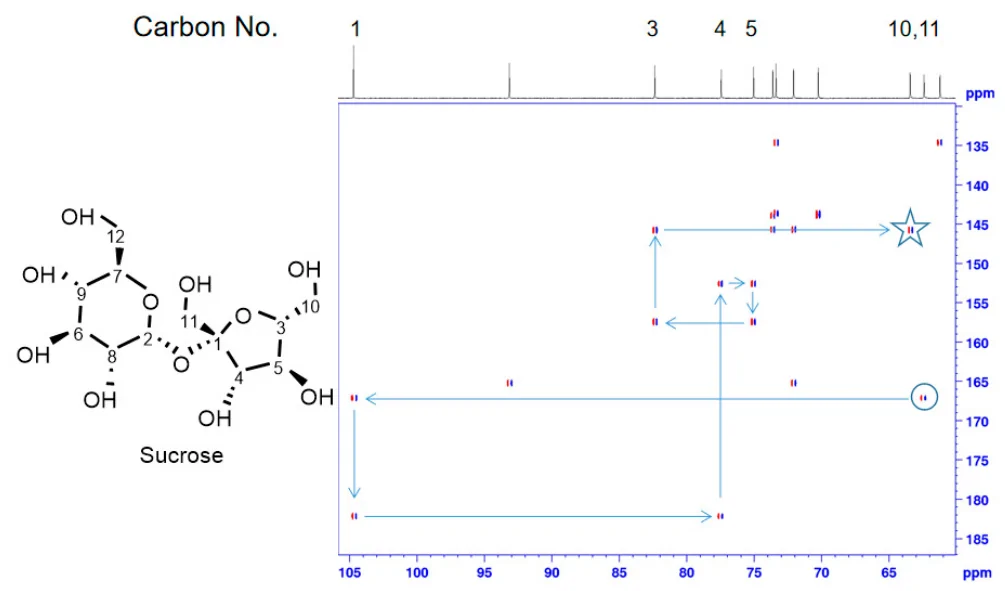
2D INADEQUATE of sucrose in D_2_O at 25 °C. Blue arrowed lines depict the carbon skeleton of scalar-coupled nuclei from C-11 (marked by a blue circle) to C-10 (marked by a blue star).

To visually illustrate the 2D INADEQUATE principles, Figure 1 presents a representative 2D INADEQUATE spectrum of sucrose acquired with the standard pulse sequence. In the direct (F_2_) dimension, directly bonded ^13^C–^13^C pairs appear as antiphase doublets (one positive and one negative peak) separated by their J_CC_. In the F_1_ dimension, the cross-peaks appear at the DQ frequency, which corresponds to the algebraic sum of the chemical shifts of the two coupled carbons. Mapping the carbon skeleton of the right ring of sucrose is shown. Starting from the antiphase doublets of carbon 11 (circled), trace horizontal connectivity to carbon 1. Next, follow the vertical connectivity of carbon 1 to a second DQ frequency, and then continue along horizontal connectivity to carbon 4. Repeat this pattern until the antiphase doublets of carbon 10 (marked by a star) are assigned.

Despite its conceptual elegance and definitive structural information, the ^13^C–^13^C 2D INADEQUATE experiment suffers from a fundamental limitation: extreme insensitivity. Due to the low natural abundance of ^13^C (approximately 1.1%), the probability of finding two adjacent ^13^C atoms in a molecule is only about 1 in 8264 C-C bonds. For a molecule containing *N* C-C bonds, the expected number of detectable ^13^C–^13^C pairs at natural abundance is *N*/8264. For example, a linear 30-carbon molecule possessing 29 C-C bonds yields only ~0.0035 detectable pairs per molecule, underscoring the severe sensitivity limitation. This statistical rarity means that the vast majority of C-C bonds in a typical sample do not contain the required ^13^C–^13^C pairs to generate a detectable INADEQUATE signal. Consequently, conventional ^13^C–^13^C 2D INADEQUATE experiments require extensive signal averaging, often necessitating days or even weeks of NMR time to achieve adequate signal-to-noise ratios (S/N). By comparison, a standard HSQC experiment on the same sample detects all ^1^H–^13^C pairs in minutes, and an HMBC provides two- and three-bond ^1^H–^13^C correlations within a comparable timeframe. This disparity explains why 2D INADEQUATE has historically been regarded as a technique of last resort, deployed only when HSQC and HMBC data prove ambiguous or insufficient.

To address this critical sensitivity limitation, several innovative approaches have been developed. Among the advancements is the Insensitive Nuclei Enhanced by Polarization Transfer-INADEQUATE (INEPT-INADEQUATE) experiment, which combines the principles of insensitive nuclei enhanced by polarization transfer with the standard 2D INADEQUATE sequence. This hybrid approach leverages the higher gyromagnetic ratio of protons to enhance the ^13^C signal intensity before initiating the 2D INADEQUATE sequence. Specifically, INEPT-INADEQUATE employs a polarization transfer step from protons to carbons, effectively transferring the greater polarization of protons (which have a natural abundance of nearly 100%) to the ^13^C nuclei. The sensitivity increase depends on the τ_1_ and τ_2_ in the INEPT pulse sequence, which are the INEPT polarization-transfer and refocusing delays, as well as the C-H coupling constant (J_CH_) [3].

Parallel to the development of INEPT-INADEQUATE, other methodological improvements have also enhanced the practical utility of the INADEQUATE experiment. The INADEQUATE CR (composite refocusing) variant, for instance, improves sensitivity by selectively transferring DQC to only one line of each doublet, effectively doubling the signal intensity compared to conventional INADEQUATE. Methodological variants such as J-compensated INADEQUATE (J-INADEQUATE) have been developed to mitigate J-mismatches and improve sensitivity. Furthermore, the integration of adiabatic pulses has mitigated off-resonance artifacts.

The INADEQUATE technique has found diverse applications across multiple domains of chemical research. In natural product chemistry, it has proven invaluable for elucidating complex structures where conventional methods fail. Similarly, in metabolomics, the technique has successfully identified carbon networks for known metabolites such as proline. In polymer science, it has enabled detailed characterization of regio-irregular structures, assigning peaks corresponding to carbons in head-to-tail (H-T), head-to-head (H-H), tail-to-tail (T-T) sequences, and various inversion patterns.

Despite these diverse applications, it is important to acknowledge that 2D INADEQUATE remains a specialist technique that relatively few practicing organic chemists have directly deployed. A systematic bibliometric analysis of its prevalence across the peer-reviewed literature is beyond the scope of this revision; nevertheless, some qualitative contextualization is useful. In natural-product structure elucidation, INADEQUATE has typically been reserved for cases in which HMBC or HSQC ambiguity persists—molecules rich in quaternary carbons, fully substituted aromatic rings, or severely overlapping resonances—and its use has increased modestly since cryogenic probe (cryoprobe) technology became more widely available from around 2005 onward. In polymer science, the technique has been applied more selectively, primarily for regioerror (RE) and tacticity assignments in polyolefins, where the severe ^13^C chemical-shift overlap in 1D spectra leaves few viable alternatives. The emergence of metabolomics applications represents a newer and growing area that may further broaden the technique’s user base. Finally, we note that the ^1^H-detected 1,1-ADEQUATE and 1,*n*-ADEQUATE variants have seen somewhat broader adoption in synthetic organic laboratories, owing to their ^1^H detection and correspondingly lower sample requirements, although they cannot detect quaternary–quaternary carbon pairs.

Despite these advances, the INADEQUATE experiment continues to evolve in response to emerging challenges and technological innovations. Recent developments include the application of artificial intelligence (AI) to enhance data quality, the integration of isotopic enrichment strategies to boost signal intensity, and the refinement of phase-cycling schemes to suppress rapid-pulsing artifacts that arise from incomplete longitudinal relaxation between scans. As cryoprobe technology continues to improve and computational methods for data processing advance, the once-prohibitive 2D INADEQUATE experiments are becoming increasingly manageable, bringing us closer to the realization of routine structural determination from milligram quantities of unknown compounds.

Before surveying the applications in detail, it is instructive to position 2D INADEQUATE relative to the principal alternative experiments for establishing C-C connectivity. HMBC provides ^2^J_CH_ and ^3^J_CH_ correlations but cannot confirm direct C-C bonds and is prone to ambiguity in proton-deficient molecules. HSQC-TOCSY can relay connectivity through proton networks but fails when proton chains are interrupted by heteroatoms or quaternary centers. LR-HSQMBC extends the HMBC range but still relies on proton observability. The ^1^H-detected ADEQUATE family (1,1-ADEQUATE, 1,*n*-ADEQUATE) offers much better sensitivity but is inapplicable to quaternary–quaternary pairs and suffers from limited ^1^H resolution. 2D INADEQUATE is the only experiment that directly and unambiguously maps every ^1^J_CC_ bond—including quaternary–quaternary connections—without reliance on protons. A decision-tree summarizing when each technique is most appropriate is provided in Section 7 (Figure 10).

While 2D INADEQUATE has also made a significant impact on solid-state NMR [4,5], this discussion emphasizes its importance in liquid-state NMR research. Earlier reviews on liquid-state 2D INADEQUATE appeared in 2002 (Buddrus and Lambert) [6] and 2010 (Uhrín) [7]. Table 1 summarizes the scope, coverage, and limitations of these prior reviews and highlights the unique contributions of the present work. In brief, the 2002 review provides a methodological overview including ^13^C–^13^C, ^29^Si–^29^Si, and ^183^W–^183^W connectivities; the 2010 review focuses on pulse-sequence theory with studies up to 2009. Neither review covers the application-rich period after 2010, Dynamic Nuclear Polarization-INADEQUATE (DNP-INADEQUATE), or AI-assisted spectral processing. In contrast, the present review compiles over 100 application entries across natural products, synthetic molecules, mixtures, oligomers, polymers, fullerenes, and metabolomics, with detailed experimental parameters and practical guidance. It also highlights foundational developments that have demonstrated enduring relevance.

## 2. Applications of Basic 2D INADEQUATE

The following examples are organized into four categories according to the nature of the sample or system under investigation, enabling readers to quickly locate applications most relevant to their own research.

### 2.1. Small Molecules’ Examples

This subsection surveys applications in which 2D INADEQUATE provided definitive C-C connectivity evidence for individual compounds—ranging from natural products and synthetic intermediates to fullerenes and biosynthetic tracers—typically in cases where conventional HSQC/HMBC data proved ambiguous due to quaternary carbons, proton deficiency, strong coupling, or severe signal overlap.

#### 2.1.1. Natural Products, Metabolites, and Biosynthetic Tracers

This subgroup highlights applications in the de novo structural elucidation, stereochemical assignment, and biosynthetic pathway tracing of biologically derived molecules.

1. Bis-nor-diterpene. Solidified the structural elucidation of the compound, including the axial configuration of C-17, inferred from its deshielded chemical shift (28.0 ppm) influenced by the C-8 oxygen bond [8].
2. Camphor. Provide carbon connectivities [9].
3. Vitamin D_3_. Unambiguous assignment of all 27 carbon chemical shifts. This resolved ambiguities in prior assignments (e.g., C-5/C-10, C-11/C-15, C-20/C-22) by directly mapping skeletal connectivities. The experiment, conducted using 0.02 M Cr(acac)_3_ to reduce relaxation times, detected 22 of 29 total connectivities [10].
4. Harman. Confirmed the bond between the methyl carbon C-14 and aromatic carbon C-3. The aromatic region expansion revealed most connectivities, except for the strongly coupled AB system of carbons C-8 and C-9, which could not be resolved in the 2D spectrum due to overlapping signals [11].
5. Brevetoxin B (BTX-B) biosynthesized from sodium [1,2-^13^C_2_] acetate. Enabled the identification of 14 acetate units within the complex polycyclic ether structure of BTX-B using only 1.5 mg of sample. 2D INADEQUATE revealed contiguous carbon pairs (e.g., C-8/C-9) with distinct chemical shifts (e.g., ~35 ppm for C-9 and ~80 ppm for C-8), allowing clear differentiation of correlated carbons in the spectrum. Cross-peaks for most acetate-derived connectivities were observed, except in regions with prolonged T_1_. The doubly labeled BTX-B served as an ideal model for biosynthetic studies, confirming the origins of all 50 carbons via [1-^13^C], [2-^13^C] acetate, and methyl-^13^C-methionine labeling. This approach resolved assignments for all carbons and highlighted the non-polyketide nature of the ladder-like oxacyclic backbone, revealing unique biosynthetic patterns such as six m-m moieties and extended methyl branches [12].
6. Erythromycin A. Key assignments included A-2 (44.9 ppm), A-8 (44.77 ppm), A-7 (38.57 ppm), A-4 (39.53 ppm), and A-10 (38.33 ppm), confirmed via coupling patterns with neighboring carbons. Methyl groups (e.g., A-2 Me at 18.26 ppm, A-8 Me at 16.23 ppm, and A-12 Me at 15.71 ppm) were definitively assigned through correlations with their respective aglycone carbons [13].
7. Hexahydrocannabinol (HHC). Mapped aliphatic carbon pairs (excluding C-6), revealing critical connectivities such as C-6–C-6a, C-6–α-CH_3_, C-6–β-CH_3_, and C-10a–C-10b. By analyzing these correlations, the aliphatic ring skeleton of HHC was unambiguously reconstructed [14].
8. Sesquiterpene lactone. Resolved complexities arising from overlapping methylene signals in conventional 1D NMR, establishing the molecular framework of the germacranolide structure [15].
9. Patellazole C. Provided clear evidence for the sequence C15–C16–C17–C18, confirming the extension of the carbon chain to C23 and thereby defining the 24-membered macrolide ring structure. Also supported the identification of C38–C2–C3, which validated the placement of the ester carbonyl at C1 (171.36 ppm). Additionally resolved the connectivity of the side chain, including the α-methyl-β-hydroxy butyrate moiety attached at C2 [16].
10. Aranorosin. Unambiguously established the attachment of heteroatoms (oxygen) to the spiro carbon (C-7) and defined the spatial arrangement of two oxirane rings and a hemiacetal group. Confirmed that four carbons—two in each oxirane ring—exhibited direct bond ^1^J_CH_ values (183.0 and 184.2 Hz), typical of oxiranes, distinguishing them from methinyloxy carbons [17].
11. Dammarane triterpenes. Analysis of 20(S)-hydroxydammaran-3-one revealed unambiguous correlations for carbons C-12 (δ 27.3 ppm), C-16 (δ 24.5 ppm), and C-21 (δ 25.4 ppm). Similarly, for dammaran-20(S)-ol, the data confirmed revised assignments for C-12 (δ 27.6 ppm), C-16 (δ 24.8 ppm), and C-21 (δ 25.6 ppm) [18].
12. Grayanotoxin I. Enabled unambiguous mapping of the tetracyclic andromedane skeleton [19].
13. Abietic acid and maleopimaric acid. For maleopimaric acid, 19 of 20 aliphatic connectivities were resolved, with the missing C-1–C-10 correlation attributed to strong AB coupling. The technique, optimized for sp^3^-sp^3^ carbon pairs and enhanced by Cr(acac)_3_ to reduce T_1_, enabled corrected assignments for previously ambiguous resonances. All 20 carbon signals in abietic acid were unambiguously assigned [20].
14. Cylicodiscic acid. The ^13^C–^13^C connectivity data confirmed the carbon skeleton of the molecule and unambiguously assigned the positions of the CH_2_OH and CO_2_H groups on the angular carbons C-14 and C-17, respectively. This established the structure of cylicodiscic acid as 3β,27-dihydroxylup-20(29)-en-28-oic acid, resolving prior uncertainties about the regiochemistry of the hydroxyl and carboxylic acid groups [21].
15. Acrihellin and hellebrigenin. For acrihellin, it revealed all C-C correlations within the steroid backbone (excluding C-1–C-2), including critical distinctions between poorly resolved signals of C-4 (35.97 ppm) and C-6 (36.07 ppm). For hellebrigenin, it confirmed previously reported carbon assignments, particularly demonstrating upfield shifts for C-6 (36.16 ppm) and C-7 (23.77 ppm) relative to acrihellin [22].
16. Astellatol, a new and unusual Sesterterpene. 2D INADEQUATE confirmed the connectivity of the tricycloundecane ring system (C-1 and C-9–C-15) fused to a six-membered and a five-membered ring [23].
17. Triterpene 3β,6β,16β-trihydroxylup-20(29)-ene. Resolving key carbon sequences such as C-25→C-10→C-1→C-2→C-3, C-9→C-11→C-12→C-13, and C-18→C-19→C-21→C-22 [24].
18. Jatamansic acid. Revealed inconsistencies in the previously proposed structure, which was originally assigned based on degradation studies. Demonstrated the correct structure features an α,β-unsaturated carboxylic acid chromophore within a seven-membered ring. Key C-C correlations, such as C-2 linked to C-8a and C-3, C-3a connected to C-5 and C-12, and C-7 correlated with C-6 and C-8a, supported the revised framework. This structural reassignment aligned with subsequent chemical degradation results, confirming that the earlier assignment of a five-membered ring system was incorrect [25].
19. Cyclic 2,3-diphosphoglycerate (cDPG). Revealed no correlation between carbonyl carbon signals (184, 182.5, and 176 ppm) and the CH carbon (79 ppm) of cDPG, indicating that carbohydrate degradation does not significantly contribute to label backflow into cDPG. Additionally, no major ^13^C–^13^C coupled species were observed in the carbohydrate region (e.g., 67–74 ppm) of the spectra, except transient peaks tentatively assigned to phosphorylated C_3_ intermediates linked to macromolecules. These findings suggest that cDPG is part of a novel metabolic branch in gluconeogenesis, distinct from classical glycolytic pathways, and does not derive its label from extensive carbohydrate breakdown [26].
20. Dicranin (Z,Z,Z-octadeca-6-yne-9,12,15-trienoic acid). The experiment utilized a single four-pulse sequence with a last pulse of ~125° to suppress unwanted image peaks. Optimized for a one-bond coupling constant of 62 Hz. The experiment required ~135 h of total NMR time. Analysis confirmed the positions of the triple bond (C-6/C-7) and double bonds (C-9/C-10, C-12/C-13, C-15/C-16). Challenges arose from overlapping signals in strongly coupled AB systems (e.g., C-6/C-7 and C-13/C-16), where only inner lines were resolved. The near-degenerate signals of C-14 and C-11 required high horizontal digital resolution for differentiation [27].
21. Ambigol A. Mapped three aromatic fragments (I, II, and III) and their interconnections. For instance, fragment I (positions 1″–6″) was resolved through sequential correlations (C-1″→C-6″→C-5″→C-4″→C-3″→C-2″), while fragment II (positions 1–6) and fragment III (positions 1′–6′) were linked via a C-1–C-1′ bond. The experiment confirmed the tricyclic structure and clarified the positions of hydroxyl and chlorine substituents [28].
22. Obscurinervine. Enabled the detection of 19 out of 23 possible ^13^C–^13^C connectivities, allowing unambiguous assignment of 20 carbon atoms in the molecule. The remaining carbons could not be definitively assigned, likely due to overlapping signals or insufficient spectral resolution [29].
23. Shikometabolin A. Confirmed the sequences of quaternary carbons, which are challenging to resolve with conventional NMR approaches. By analyzing correlations, the experiment revealed key connections such as carbon A (C-1′) linked to X (C-9′) and M (C-2′), and carbon B (C-5) connected to Y (C-10) and N (C-6). These findings were critical in establishing the dimeric structure of shikometabolin A, including the spatial arrangement of rings and the identification of bridging carbons (e.g., C-11′ linking partial structures). Utilized a coupling constant of J_CC_ = 60 Hz and required a 36 h NMR time to achieve sufficient sensitivity for complex quaternary carbon networks [30].
24. Aldopentoses. For D-xylopyranose, it distinguished closely spaced resonances (e.g., C-4α and C-4β) based on signal intensity differences between anomers. However, strong ^13^C–^13^C scalar coupling (e.g., between C-2 and C-3 in L-lyxose) caused signal intensity losses and spectral artifacts, complicating assignments. For D-ribose, it assigned the β-pyranose anomer but failed for the α-pyranose and furanose forms due to low abundance and signal overlap [31].
25. Conocephalenol. Provided unambiguous evidence for the brasilane skeleton of conocephalenol, revealing its unique bicyclic carbon framework. This finding aligned with the previously reported structure of brasilenol, marking conocephalenol as the first brasilane-type compound isolated from a liverwort. 2D INADEQUATE confirmed the presence of a tetrasubstituted double bond (δ 135.9 ppm and 132.7 ppm in ^13^C NMR) and tertiary hydroxy group (δ 74.0 ppm in ^13^C NMR), which were essential for elucidating the molecule’s core architecture [32].
26. Cyclosantalic acid and epicyclosantalic acid. Revealed both acids feature a tricyclic ring system composed of a norbornane partial structure fused to a cyclopentane ring, with a propionic acid residue attached at the C-1 position. This constitutional framework was confirmed for both stereoisomers. The results established the core tricyclic skeleton critical for assigning the relative configurations of the aldehydes through subsequent Nuclear Overhauser Enhancement (NOE) and force field analyses [33].
27. Pregaleopsin. While initial 2D NMR experiments (COSY, HMQC, HMBC) provided partial structural insights, they were insufficient to fully assign all resonances due to overlapping signals and complex coupling patterns. The INADEQUATE spectrum (recorded at J = 60 Hz) clarified the complete assignment of both proton and carbon resonances. This was particularly vital for resolving ambiguities in the labdane diterpenoid skeleton, ensuring accurate stereochemical and regiochemical assignments. The authors noted that earlier literature data for pregaleopsin were either incomplete or incorrect, and the INADEQUATE analysis provided definitive evidence to rectify these discrepancies [34].
28. [1,2-^13^C]acetate-labeled andrastin A. Confirmed the connectivity of rings A, B, and C in the androstane skeleton, excluding ambiguities in the bonds involving C-8 (δ 42.8 ppm), C-14 (δ 68.6 ppm), and C-13 (δ 57.8 ppm). The technique identified key cross peaks corresponding to C-15 (δ 187.4 ppm)/C-16 (δ 114.5 ppm), C-14/C-26 (δ 171.8 ppm), and C-16/C-28 (δ 6.1 ppm), which were critical for mapping the cyclopentane ring D and its substituents [35].
29. Nucleoside analog 4-(2′-deoxy-β-D-erythro-pentofuranosyl)-7,7-dimethyl-2,4-diazabicyclo [4.2.0]octa-1,5-dien-3-one. Revealed cross-peaks corresponding to the C(4)–C(8) bond within the cyclobutane moiety. The doublet nature of these cross-peaks confirmed the presence of a ^1^J_C4,C8_ coupling constant of 27 Hz, which is consistent with the strained cyclobutane framework. Additional coupling constants, such as ^1^J_C7,C8_ (26 Hz) and ^1^J_C7,C9/C10_ (34 Hz), further validated the cyclobutane ring’s structure. This data conclusively demonstrated the formation of the 1,2-dihydrocyclobuta[d]pyrimidin-2-one core, distinguishing it from alternative structural isomers [36].
30. Sarsasapogenin. Nearly all one-bond ^13^C–^13^C couplings were identified, providing critical insights into its carbon framework. 2D INADEQUATE revealed ambiguities in three strongly coupled carbon pairs (C-12/C-13, C-6/C-7, and C-23/C-24), which could not be fully resolved due to spectral overlap [37].
31. Haliclonacyclamines A and B. For haliclonacyclamine A, secure assignments of C5 (45.4 ppm) and C7 (37.8 ppm) served as anchors, enabling unambiguous mapping of all 30 remaining carbons. In haliclonacyclamine B, corrected prior misassignments: signals at 59.2 and 37.7 ppm were reassigned to C6 and C7, respectively, aligning with haliclonacyclamine A’s core structure [38].
32. ^13^C-enriched aspen lignin. Revealed distinct cross-peaks corresponding to four types of β-O-4 arylglycerol aryl ether structures: erythro arylglycerol β-syringyl ethers (Cα/Cβ: 72.5/86.0 ppm), threo arylglycerol β-syringyl ethers (71.9/87.2 ppm), erythro arylglycerol β-guaiacyl ethers (72.2/83.5 ppm), and threo arylglycerol β-guaiacyl ethers (71.4/84.5 ppm). The enhanced sensitivity of ^13^C-enriched samples enabled precise differentiation of diastereomeric forms, underscoring the power of 2D INADEQUATE NMR in resolving lignin’s complex stereochemistry. This study demonstrated that erythro β-syringyl ethers dominate in aspen lignin, aligning with biosynthetic preferences, while β-guaiacyl ethers exhibit a balanced distribution of stereoisomers [39].
33. Baccharis oxide. By optimizing the experiment for sp^3^-sp^3^ hybridized carbon correlations and supplementing the sample with 0.03 M Cr(acac)_3_ to reduce relaxation delays, the researchers mapped the complete carbon framework. Key correlations included the signal at δ 93.8 ppm (C-10) linked to C-5 (δ 53.1 ppm), C-1 (δ 32.0 ppm), and C-9 (δ 37.3 ppm). Sequential analysis of these connectivities established the full carbon sequence, such as C-1 (δ 32.0 ppm) correlating with C-2 (δ 24.8 ppm), which further connected to C-3 (δ 84.3 ppm) [40].
34. Drimiopsin A. Confirmed the placement of methoxy groups at C-5 and C-7 and hydroxy groups at C-6 and C-8, resolving ambiguities caused by the absence of proton correlations. Similarly, in drimiopsin B, the 2D INADEQUATE data, combined with NOESY correlations, established methoxy groups at C-5, C-6, and C-7. For drimiopsin F (6), it clarified that a methoxy group occupies C-3 instead of a hydroxy group, while hydroxy groups were assigned to C-6 and C-8. These findings underscored the utility of 2D INADEQUATE NMR in mapping fully substituted aromatic rings when traditional methods (e.g., COSY, NOESY) failed to provide conclusive structural details [41].
35. An inhibitor A-74528. Verified the direct connectivities between C-6 and C-6a, as well as C-11 and C-11a, which were pivotal for confirming the highly fused polyketide structure [42].
36. Tridentorubin. Revealed a functionalized benzofuro [5,6-b]benzofuran system (designated as core A) as the central scaffold. Key connectivities included C-1–C-2–C-3–C-4–C-5–C-6–C-1, forming a bicyclic ring system. Additional correlations such as C-2–C-14′–C-15′–C-19′/C-20′, C-13′–C-14′, and C-5–C-1″–C-2″–C-3″–C-4″–C-5″–C-6″ established the fusion of aromatic rings with the geranylgeranyl side chain [43].
37. 2,3:5,6-di-O-isopropylidene-α-D-mannofuranose. Notably, 10 mg of this sample yielded a high-quality INADEQUATE spectrum in 9 h using a cryoprobe. NMR was on 500 MHz, whereas the probe diameter was not mentioned [44].
38. Tricyclocohumol. Revealed ^1^J_CC_ of 34 Hz (C1′/C2′) and 36 Hz (C3′/C3′), unambiguously confirming the tricyclic structure. These results provided definitive evidence for the carbon framework rearrangements during beer storage, enabling the identification of novel bitter compounds such as tricyclocohumol and related derivatives [45].
39. Karlotoxin-2. Revealed connections for C1–C22, C23–C33, C34–C39, C40–C53, and C58–C63. The ^13^C-enriched sample (10% ^13^C) and 600 MHz NMR with a cryoprobe enabled high-quality spectral data, critical for mapping the complex framework [46].
40. Histidine. A high-sensitivity 1.5 mm cryoprobe enabled successful application of this method to natural abundance 1.1 mg of histidine (7 μmol) dissolved in 35 μL D_2_O; a 2D INADEQUATE spectrum was acquired in 48 h with adiabatic pulses. This contrasts sharply with conventional 5 mm probes, which would require nearly two weeks for the same experiment [47].
41. Yellow chlorophyll catabolite (Ed-YCC). Enabled the unambiguous identification of four contiguous carbon fragments (F1–F4) in the structure of Ed-YCC. The experiments were run on a 800 MHz with J_CC_ = 40 and 70 Hz. These fragments included F1 (C3^2^–C3^1^–C3–C2–C1–C20–C19–C18–C17–C16–C15–C13^2^–C13^3^), F2 (C13^1^–C13–C12–C11–C10), F3 (C8^1^–C8^2^), and F4 (C8^3^–C8^4^–C8^5^). This analysis confirmed key structural features such as a 1,2-dihydroxyethyl-4-methyl-2-oxo-^1^H-pyrrole moiety in F1, a 4-carbonyl-3-methyl-^1^H-pyrrole moiety in F2, and connectivity between fragments via correlations like 13^2^-H to C-13^1^ and 15-H to C-13/C-14. Resolved ambiguities in the pyrrole ring (ring B) connectivity and confirmed the trans configuration between 13^2^-H and 15-H [48].
42. Vialisyl A (p-Terphenyl). Used a Bruker AVANCE III HD 600 MHz spectrometer equipped with a 5 mm ^13^C observe cryoprobe and employed the inadgpqfsp pulse sequence with a J_CC_ value of 50 Hz, requiring approximately 90 h of NMR time. Definitive correlations were observed between C-116.6 (C(1′,4′)) and C-130.6 (C(2′,3′)), between C-116.6 (C(1′,4′)) and C-137.0 (C(6′,5′)), and between C-116.6 (C(1′,4′)) and C-112.7 (C(1′,1’’)), with no correlations detected between C-137.0 and C-130.6 [49].
43. 6-(9′,10′-Dihydro-1′,8′-dihydroxy-3′-methoxy-6′-methyl-9′,10′-dioxoanthryl)-2′-yl-11,13-dihydroxy-9-methoxy-3-methylbenzo-[1,2-g]isochroman-1-one. Conventional ^1^H–^13^C correlation techniques provided insufficient structural information due to the high number of non-protonated aromatic carbons. Analysis of the 2D INADEQUATE data revealed that it is a heterodimer comprising two tricyclic moieties, an anthraquinone and a 3,4-dihydro-3-methylbenzoisocoumarin, rather than the previously proposed xanthone structure. The key structural insight came from the correlation between C-6 (δ_C_ 116.8 ppm) and C-2′ (δ_C_ 118.9 ppm), which established the linkage between the two constituent units. Although this junction was also suggested by HMBC correlation between H-4′ (δ_H_ 7.51 ppm) and C-6 (δ_C_ 116.8 ppm), it could not be reliably confirmed without the 2D INADEQUATE due to the lack of available HMBC correlations and neighboring non-protonated carbon atoms. Additional critical correlations included those of C-9′ (δ_C_ 190.6 ppm) with C-12′ and C-14′, and C-10′ (δ_C_ 181.1 ppm) with C-11′ and C-13′, which confirmed the anthraquinone structure and ruled out an aromatic carboxylic acid moiety. The meta-relationship between H-8 and H-10 was also definitively confirmed through correlations of both C-8 and C-10 with C-9. Bruker pulse sequence inadphsp on a Bruker Avance III 950 MHz, with J_CC_ 60 Hz to favor signals from pairs of sp^2^ carbons. The experiment required 3 days and 7 h of NMR time to achieve sufficient signal at natural ^13^C abundance [50].
44. [^13^C_2_]-labeled 5′-O-methyldioncophylline D and habropetaline A. Revealed distinct doublets for C-1 and Me-1 at 50.0 and 18.2 ppm, respectively, which were substantially larger than the corresponding peaks in non-labeled compounds. These measurements confirmed incorporation of the labeled precursor into the complete alkaloids to an extent of approximately 8%, comparable to the labeling degree observed for dioncophylline A. 2D INADEQUATE specifically established the C-C connectivities for the ^13^C_2_-labeled unit, providing conclusive evidence that the tetrahydroisoquinoline serves as the authentic coupling substrate in the biosynthetic pathway rather than its dihydroisoquinoline precursor [51].
45. Biosynthetic origin of anthracimycin. After feeding [1,2-^13^C_2_]acetate to Streptomyces sp. TP-A0875, cross-peaks in 2D INADEQUATE (optimized for J_CC_ = 45 Hz) confirmed intact acetate units at the following carbon pairs: C-1/C-2, C-3/C-4, C-5/C-6, C-7/C-8, C-9/C-10, C-13/C-14, C-15/C-16, C-17/C-18, C-19/C-20, and C-21/C-22. The C-11/C-12 pair initially failed to show a cross-peak due to strong coupling from minimal chemical shift differences (Δδ = 0.11 ppm in CDCl_3_). By switching to C_6_D_6_ (Δδ = 0.31 ppm) and adjusting the delay parameter from τ = 1/(4J_CC_) to τ = 3/(4J_CC_), the C-11/C-12 coupling was successfully detected, confirming their joint derivation from a single acetate unit. Interestingly, both C-11 and C-12 are sp3 hybridized, yet the reported coupling constant between them is 73 Hz, which is unusually high. The C-7/C-8 pair has a similar structure, and the J between them is 35 Hz [52]. The unusually large ^1^J_CC_ of 73 Hz for the C-11/C-12 pair, compared with 35 Hz for the structurally analogous C-7/C-8 pair, likely reflects differences in ring strain, hybridization perturbation, or substituent effects within the tricyclic macrolide framework. A detailed quantum-chemical analysis of this discrepancy would be of interest but is beyond the scope of this review.
46. Garlic-derived compounds. Confirmed the absence of ^13^C–^13^C correlations between C5 and C1′ as well as between C2 and C5. This evidence was critical in disproving the previously proposed structure that suggested nine contiguous carbons. As stated in the paper, these 2D INADEQUATE findings, along with supporting evidence from X-ray absorption spectroscopy and computational modeling, demonstrated that the compounds were 5-(allylsulfinyl)-3,4-dimethylthiolane-2-ols rather than the thiolanesulfenic acids that had been previously claimed [53].
47. Methylobamine. Due to the limited number of hydrogen atoms in the molecule, conventional HMBC spectral analyses proved insufficient for determining linkages to other carbon atoms. To address this limitation, ^13^C-enriched methylobamine by cultivating Methylobacterium sp. strain W-213 was prepared on a medium containing D-[^13^C_6_]glucose. The 2D INADEQUATE of this ^13^C-enriched sample revealed a critical structural feature: a linear linkage of five carbon atoms in addition to those comprising the galactopyranose moiety. This information was instrumental in establishing the complete C-C skeleton of methylobamine. The data provided definitive evidence for the connectivity between the pyranooxazin moiety and the iminoacetic acid component of the molecule, which was essential for determining the complete chemical structure [54].
48. Ascorbigen A. The experiment was conducted using a 5 mm broadband probe with J_CC_ = 50 Hz. The experiment traced C-C connectivity starting from C2 (characteristic of a COO carbon), but failed to reveal C4′-C5′ and C5-C6 connectivities due to strong coupling in these pairs (chemical shift differences of 0.66 and 0.36 ppm, respectively) [55].
49. Perisalazinic acid. A hydrogen-poor depsidone with only 4 of 18 carbons possessing C-H bonds, which made conventional NMR techniques problematic due to the extremely limited number of protons. Determined connectivity and ^1^J_CC_ values for all C-C bonds in the molecule, with the exception of overlapping signals for C5′/C6′ and ambiguous resonances at C1/C2 and/or C2/C3. These experimental ^1^J_CC_ values were then compared with density functional theory (DFT) computed values to evaluate 81 candidate structures, ultimately identifying the best-fit structure. The study demonstrated that 2D INADEQUATE-derived ^1^J_CC_ values can enable complete three-dimensional structural characterization of compounds that are challenging to analyze with traditional proton-based NMR methods, particularly for hydrogen-deficient molecules that fall under “Crews rule”, where the proton-to-heavy-atom ratio is less than two [56].
50. Chrozophoridin. The blue pigment isolated from Chrozophora tinctoria fruits. While most carbon assignments were achieved through other 2D NMR techniques (HSQC and HMBC), the chemical shift of carbon C-11 could only be definitively determined using the 2D INADEQUATE experiment. This technique revealed a direct correlation between C-11 and C-12, which was essential for proper structural assignment. The 2D INADEQUATE showed that carbon C-11 appeared at a high chemical shift value (172.6/172.3 ppm for the two atropisomers), which was consistent with the presence of a carbonyl group at this position. This information was particularly valuable since carbons C-3, C-9, and C-11 lacked directly attached protons, making their assignment impossible through standard proton-coupled techniques. The 2D INADEQUATE experiment thus provided the crucial through-bond C-C connectivity evidence needed to confirm the complete molecular structure of this historically significant medieval blue dye [57].
51. Dehydrocrebanine. A 10.4 mg sample on an 800 MHz spectrometer equipped with a cryoprobe assigned nearly all C-C connectivities despite the absence of ^13^C enrichment. The measurement, accumulated over 55 h, provided direct evidence for the structure by revealing key ^13^C–^13^C coupling correlations, including C1–C2 (141.6–145.2 ppm), C2–C3 (145.2–106.8 ppm), C3–C3a (106.8–127.6 ppm), C3a–C4 (127.6–30.8 ppm), C4–C5 (30.8–50.6 ppm), C6a–C6b (144.1–118.3 ppm), C6b–C3a (118.3–127.6 ppm), C6a–C7 (144.1–93.9 ppm), C7–C7a (93.9–129.5 ppm), C7a–C8 (129.5–141.5 ppm), C9–C10 (150.2–108.5 ppm), C10–C11 (108.5–123.4 ppm), C11–C11a (123.4–118.6 ppm), C11a–C7a (118.6–129.5 ppm), C11b–C6b and C11a–C11b (overlapped signals at 117.4–118.3 ppm), and C11b–C1 (117.4–141.6 ppm). This comprehensive carbon skeleton mapping confirmed the correct structure of dehydrocrebanine and allowed revision of previously misassigned NMR data [58].
52. Metabolites such as proline, DHPS, β-1,3-glucan, choline, and glutamate. The 2D INADEQUATE proved especially valuable in resolving overlapping signals in crowded spectral regions like sugars, where ^1^H NMR typically suffers from severe peak overlap [59].

#### 2.1.2. Synthetic Molecules, Fullerenes, and Organometallics

This subgroup highlights the use of 2D INADEQUATE to confirm novel synthetic scaffolds, resolve symmetric or strongly coupled systems, and map entirely proton-free carbon cages where heteronuclear proton-detected experiments are entirely inapplicable.

**Figure 2:**
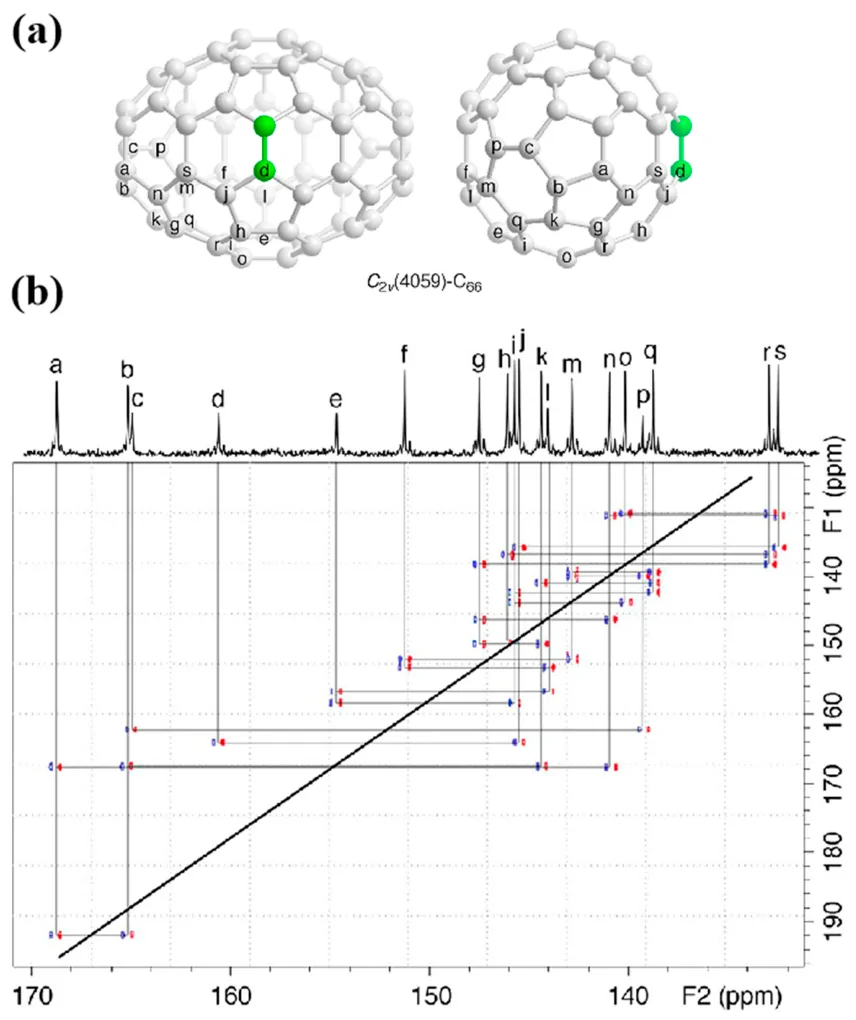
(**a**) The true structure of Sc_2_@C_66_. (**b**) 2D INADEQUATE NMR spectrum of ^13^C enriched Sc_2_@C_66_ in CS_2_. The thin black lines: ^13^C–^13^C coupling correlations. Reproduced from Ref. [88] with permission from the American Chemical Society.

1-methylazulene and 5-methylazulene. Provided a comprehensive coupling matrix, including previously inaccessible values such as those across the central C-9–C-10 bond in azulene [60].
3.1- and 2-methylnaphthalene. Unambiguous assignment of C-C connections [61].
1-methoxy-endo-tetracyclo [6.3.0.0^2,11^.0^3,7^]undec-9-ene. The experiment revealed the cyclopropane carbons C-10/11 (δ 36.6 ppm, J_CH_ = 160.1 Hz) and C-2 (δ 38.6 ppm, J_CH_ = 159 Hz), which were coupled to C-3 (δ 48.9 ppm). Olefinic C-9 (δ 134.6 ppm) showed connectivity to C-8 (δ 54.9 ppm), which further linked to C-7 (δ 60.3 ppm) and C-1 (δ 95.6 ppm). The methoxy group’s carbon appeared at δ 55.9 ppm, while AB-type splitting patterns and strong couplings complicated some connectivities [62].
2-substituted adamantanes. Contour plots of 2-phenyladamantane and 2-bromoadamantane revealed distinct satellite peaks separated by DQ frequencies, enabling unambiguous linkage of δ resonances to their corresponding γ signals [63].
Imipramine. Previous assignments of non-protonated aromatic carbons (C_1a_ and C_4a_) were reversed, with C_4a_ identified as the most downfield peak due to the inductive effect of the nitrogen atom [64].
Synthetic chlorin H_2_(OEC). Confirmed that the signal at 167.72 ppm, assigned to the α-pyrroline carbons, was connected to the β-pyrroline carbons resonating at 54.58 ppm. This connectivity validated the reassignment of the α-pyrroline carbons to a deshielded region (150–175 ppm), contrasting earlier incorrect assignments [65].
4a,7-dihydropleiadiene. Two iterations of the technique were employed: the first covered the full carbon spectral window (15,151 Hz) with a 5 ms 1/(4J) delay, while the second focused on sp^2^-hybridized carbons with a 1748 Hz sweep width in the F_2_ dimension. To enhance resolution for quaternary carbons, Cr(acac)_3_ was added to reduce relaxation times. The experiments revealed methylene carbon B linking to quaternary carbon Q3 (131.81 ppm) and the olefinic carbon at 128.09 ppm (E). Further correlations, including Q1–F1/F3/Q2, Q2–A/C/D1, and Q3–B/D2/E, enabled unambiguous mapping of the trisubstituted aromatic ring and olefinic framework [66].
4-vinylcyclohexene oxide. Revealed the presence of four stereochemical isomers (A–D) by mapping ^13^C–^13^C connectivities along the carbon skeleton [67].
Organoplatinum complex (ɳ^5^-MeCp)PtMe_3_. Revealed coupling between the ipso carbon (114.17 ppm) and the carbon at 91.41 ppm, as well as between the two methine carbons (91.41 and 95.68 ppm). These correlations confirmed that the 91.41 ppm signal corresponds to the α-carbon relative to the methyl substituent, overturning prior spectroscopic assignments [68].
C_60_(OsO_4_)(4-tert-butylpyridine)_2_. Resolved 17 distinct carbon types in the C_60_ framework, with four carbons lying on approximate mirror planes (types 1, 8, 13, and 17) exhibiting half-intensity peaks. Connectivities derived from cross-peaks (e.g., a–q, b–h, c–d, e–i, k–q, m–p) enabled unambiguous assignments of carbon types and confirmed the cup-shaped topology of the osmylated C_60_ segment [69].
Fullerene C_70_. Showed four distinct carbon connectivities, with resonances in a 10:10:20:20:10 intensity ratio, confirming the assignment of five chemically unique carbon types. Crucially, the connectivity between two low-intensity resonances at 150.8 ppm and 147.8 ppm identified the polar end-cap carbon and validated the remaining assignments [70].
Butadiene trimers. Confirmed the presence of one isolated double bond and three conjugated double bonds in the trimers, which adopt a dodeca-2,4,6,10-tetraene structure [71].
3-aryl-4-cyanosydnones. By detecting C-C connectivities within the phenyl group, it unambiguously identified the resonance signals of C(2′)/C(6′) at 121.7 ppm and C(3′)/C(5′) at 130.9 ppm. The symmetrized 2D INADEQUATE spectrum revealed correlations between these carbons and the quaternary C(1′) at 134.7 ppm, confirming their positions in the aromatic ring. Resolved inconsistencies in prior studies, particularly distinguishing para/meta carbons and validating the electronic structure of sydnones. The technique also highlighted the insensitivity of meta carbons (C(3′)/C(5′)) to substituent effects (<2 ppm shift), enabling reliable assignment of ortho and para carbons. These findings supported the chain-conjugated structure of sydnones, emphasizing the role of exocyclic oxygen (O(6)) in conjugation and the positive charge at N(3) [72].
Pentacyclic pinocarvone dimer. Resolved overlapping signals in the aliphatic region (δ 20.5–45.1 ppm), distinguishing quaternary carbons (e.g., δ 40.1 and 38.9 ppm) and their connectivities to methylene/methine groups (e.g., δ 43.9, 32.9, 25.9, 27.3, and 22.1 ppm). Provided a complete carbon framework assignment, overcoming challenges posed by hindered signal dispersion and stereochemical complexity in this Diels–Alder dimer [73].
Hexanoic acid and hexadecanoic acid. Revealed correlations between carbon pairs (e.g., C1-C2, C2-C3, w2-w1) and confirmed molecular skeletons, while resolving subtle differences in J values arising from variations in hybridization states and bond angles. For instance, the significantly larger J ^13^C–^13^C for the C1-C2 (carboxylic end) bond compared to other methylene/methyl pairs highlighted differences in electronic environments [74].
(E)-cyclodec-5-enone. Revealed correlations between olefinic and aliphatic carbons, specifically linking δ 134.4 ppm (C-6) to δ 34.2 ppm (C-5) and δ 131.6 ppm (C-7) to δ 33.3 ppm (C-8). However, poor resolution in the aliphatic region necessitated a follow-up experiment focused solely on the aliphatic range (15–50 ppm). This allowed assignment of C-5 and C-6 connectivity based on their coupling to four and three aliphatic carbons, respectively [75].
Tricyclohexylmethane. The signals at 26.77 ppm and 54.40 ppm were identified as the central carbon and the 4-position carbons of the cyclohexyl rings, respectively, due to their unique connectivity to a single equivalent carbon group. Empirical chemical shift calculations corroborated the assignment of the 26.77 ppm signal to the 4-position carbons. The remaining signals (37.86, 32.28, and 27.21 ppm) were assigned to the 1′, 2′, and 3′ positions of the cyclohexyl rings, respectively, based on the connectivity patterns. Notably, neighboring carbons in the cyclohexyl rings appeared at adjacent positions in the ^13^C NMR spectrum, reflecting their spatial proximity. This analysis confirmed the conformational flexibility of 2 at room temperature, with rapid equilibration of the three cyclohexyl rings and equivalent 2/6 and 3/5 carbon pairs on the NMR timescale [76].
Chromophore (aglycon) portion of FD-594. By analyzing a [1,2-^13^C_2_] acetate-labeled derivative of FD-594, the experiment confirmed that all carbons in the aglycon skeleton, except for the methoxy group and C-8a, exhibited triplet signals due to intact acetate unit incorporation. Provided unambiguous mapping of the polyketide backbone derived from 14 acetate units. This analysis revealed the loss of one carbon atom during biosynthesis and validated the connectivity of the pyrano [4′,3′:6,7]naphtho [1,2-b]xanthene core structure. Coupling constants from the labeled acetate feeding experiments further supported the proposed biosynthetic pathway, demonstrating the utility of 2D INADEQUATE in resolving complex carbon frameworks enriched with isotopic labels [77].
1,1,2,4,4-pentachlorobuta-1,3-diene. A unique ^1^J_CC_ correlation of 95 Hz was observed between C-3 (unambiguously assigned at 123.17 ppm) and either C-2 or C-4. This data, combined with coupling constant analysis, resolved ambiguities in carbon assignments, confirming C-4 at 129.65 ppm and C-2 at 123.54 ppm through exclusion and splitting pattern analysis [78].
1,16-dialkyl-1,16-dihydro[60]fullerene. The structure was assigned [79].
Hexaanionic fullerene C_70_^6-^. Revealed four key interactions between carbon signals, enabling the assignment of all five distinct carbon environments in C_70_^6-^. Specifically, the correlation between signals at δ 133.6 ppm (10 carbons) and δ 158.3 ppm (10 carbons) confirmed the connectivity of carbons at the poles (a) and equatorial regions (b) of the ellipsoidal C_70_^6-^ structure. Further analysis of cross-peaks led to the full assignment: carbons a (δ 133.6 ppm), b (δ 158.3 ppm), c (δ 152.3 ppm), d (δ 138.1 ppm), and e (δ 149.8 ppm). 2D INADEQUATE provided critical insights into the charge distribution and magnetic properties of the fullerene anion, revealing site-specific deshielding and shielding effects linked to the localization of added electrons on polar pentagonal rings [80].
[La@C_82_-A]^-^. Enabled definitive mapping of bond connectivity and complete assignment of all 24 carbon atoms in the fullerene cage, a first for fullerenes beyond C_60_ and C_70_. By correlating ^1^J_CC_ with bond lengths and bond orders, the study revealed that the 5,6- and 6,6-ring-fusion bonds in La@C_82_-A exhibit overlapping bond length and coupling constant distributions, contrasting with the distinct single-/double-bond character observed in empty fullerenes like C_60_ and C_70_. This overlap suggests a less clear differentiation between single- and double-bond character in La@C_82_-A, attributed to La-induced electron transfer affecting the antibonding orbitals of the carbon cage. Additionally, the position of the La atom was validated through T_1_ measurements, which correlated with calculated La–C distances, confirming La’s location near the closest carbon atoms [81].
E-edge-[60]fullerenylmethanodihydropyrrole. Identified sequential four-bond connectivities between sp^3^ carbons at δ 69.8 ppm (malonate site, C-16/17) and δ 82.9 ppm (dihydropyrrole site, C-1), validating the e-edge addition. Additionally, the Cs-symmetry was established through half-intensity resonances for fullerenyl sp^3^ carbons (C-52 and C-60) and equivalent benzylic methylene protons. These findings were corroborated by HMBC correlations, such as a strong 3-bond correlation from ortho-phenyl protons to the dihydropyrrole quaternary carbon (δ 95.9 ppm, Cα) and weaker 4-bond correlations to C-9 (δ 81.2 ppm) [82].
[Ce@C_82_]^-^. Enabled complete assignment of all carbon chemical shifts in [Ce@C_82_]^-^. This assignment was critical for analyzing lanthanide-induced NMR shifts, particularly the dominant pseudocontact shift (δpc) contributions arising from the paramagnetic Ce atom. Revealed 28 cross-peaks corresponding to 30 total bonds, with missing peaks attributed to signal broadening or overlapping chemical shifts (e.g., C(2)–C(3) and C(18)–C(19)). These results provided a foundation for determining the off-centered position of the Ce atom near a hexagonal ring of the C_82_ cage [83].
7,7a,13,14-tetrahydro-6H-cyclobuta[b]pyrimido [1,2-a:3,4-a′]diindole. Analyzed with a 5 mm DCH cryoprobe in a 600 MHz NMR; the NMR time was 66 h using a 70 mg sample. Key observations included correlations between indolic carbon C-15 (δ 98.4 ppm) and C-15a (δ 128.9 ppm)/C-14a (δ 135.3 ppm), which established the ethylene moiety (CH_2_-CH_2_) linked to a nitrogen atom. Additionally, the quaternary carbon C-5a (δ 83.3 ppm) was identified as the central junction between two nitrogen atoms, with further correlations to methine (C-7a, δ 48.1 ppm) and methylene (C-6, δ 34.2 ppm) carbons, confirming the cyclobutane ring [84].
1-phenylpiperazine. Revealed clear correlations among the following carbon atoms: C1–C2, C3–C4, C7–C8–C12, C8–C12–C11–C13, and C11–C13–C10. These correlations confirmed the structural framework, particularly the connectivity between the piperazine ring (C1–C4) and the phenyl group (C7–C13), as well as the internal bonding patterns within the aromatic ring [85].
Compounds 3 (N-(t-butyloxycarbonyl)-2-aminobenzo[d]isothiazol-3(2H)-one) and 4 (N-(t-butyloxycarbonyl)-2-aminoisoindolin-1-one). For compound 3, it confirmed the aromatic ring correlations starting from unambiguous C3 (carbonyl group at δ 164.9 ppm), revealing that C3a (δ 122.0 ppm) correlates with C4 (δ 127.3 ppm) and a quaternary carbon at δ 140.4 ppm (assigned as C7a). This enabled differentiation of C3a and C7a, which were indistinct in HMBC spectra. For compound 4, it confirmed C3 (δ 167.6 ppm) as a quaternary carbon linked to C3a (δ 130.3 ppm) and validated the aromatic ring connectivity network. These results resolved uncertainties in long-range coupling patterns observed in HMBC and HSQC experiments, particularly for non-protonated carbons [86].
Enaminone condensation–cyclization reaction product. 2D INADEQUATE was performed, ^1^J_CC_ = 50 Hz. Confirmed the substitution pattern of the heterocycle, specifically identifying the R group at C-2 and resolving ambiguities arising from nearly identical ^1^H and ^13^C NMR spectra of regioisomers. While the quaternary carbon C-8 (δ 67.9 ppm) lacked direct ^1^J_CC_ correlations, the remaining framework—including the phenyl group and heterocyclic core (e.g., C-2, C-3, C-4, C-5)—was mapped unambiguously. This eliminated uncertainties from HMBC data limitations, such as overlapping ^2^J and ^3^J couplings [87].
Sc_2_@C_66_. The 2D INADEQUATE provided critical structural insights into the Sc_2_@C_66_ endohedral fullerene (Figure 2). Analysis of the ^13^C-enriched sample revealed 21 clear cross-peaks that established the bond connectivity within the C_66_ cage. The data identified two contiguous hexagons with sequences e-l-f-m-q-i-e and q-i-o-r-g-k-q, along with an adjacent pentagon (g-k-b-a-n-g) connected to the latter hexagon. Additionally, a sequential bond connectivity pattern f-m-p-c was observed. The carbons labeled c, d, e, l, and p exhibited half-intensity signals, indicating their positions on mirror planes. This information allowed researchers to eliminate six of the eight possible C_2v_-C_66_ isomers (C_2v_(315), C_2v_(344), C_2v_(4152), C_2v_(4155), C_2v_(4348), and C_2v_(4349)), definitively ruling out the previously proposed C_2v_(4348)-C_66_ structure. Although the technique could not distinguish between the remaining candidates C_2v_(3441)-C_66_ and C_2v_(4059)-C_66_ due to identical bond connectivity (as the former can be generated from the latter via a Stone–Wales transformation), it conclusively demonstrated that Sc_2_@C_66_ must possess one of these two structures rather than the originally reported isomer [88].

### 2.2. Oligomers and Polymers Examples

This subsection examines applications in which 2D INADEQUATE resolved regioerrors, tacticity, comonomer distributions, and chain-end structures in macromolecules-structural questions that are largely inaccessible to conventional 1D ^13^C NMR owing to severe spectral overlap in the polymer backbone.

**Figure 3:**
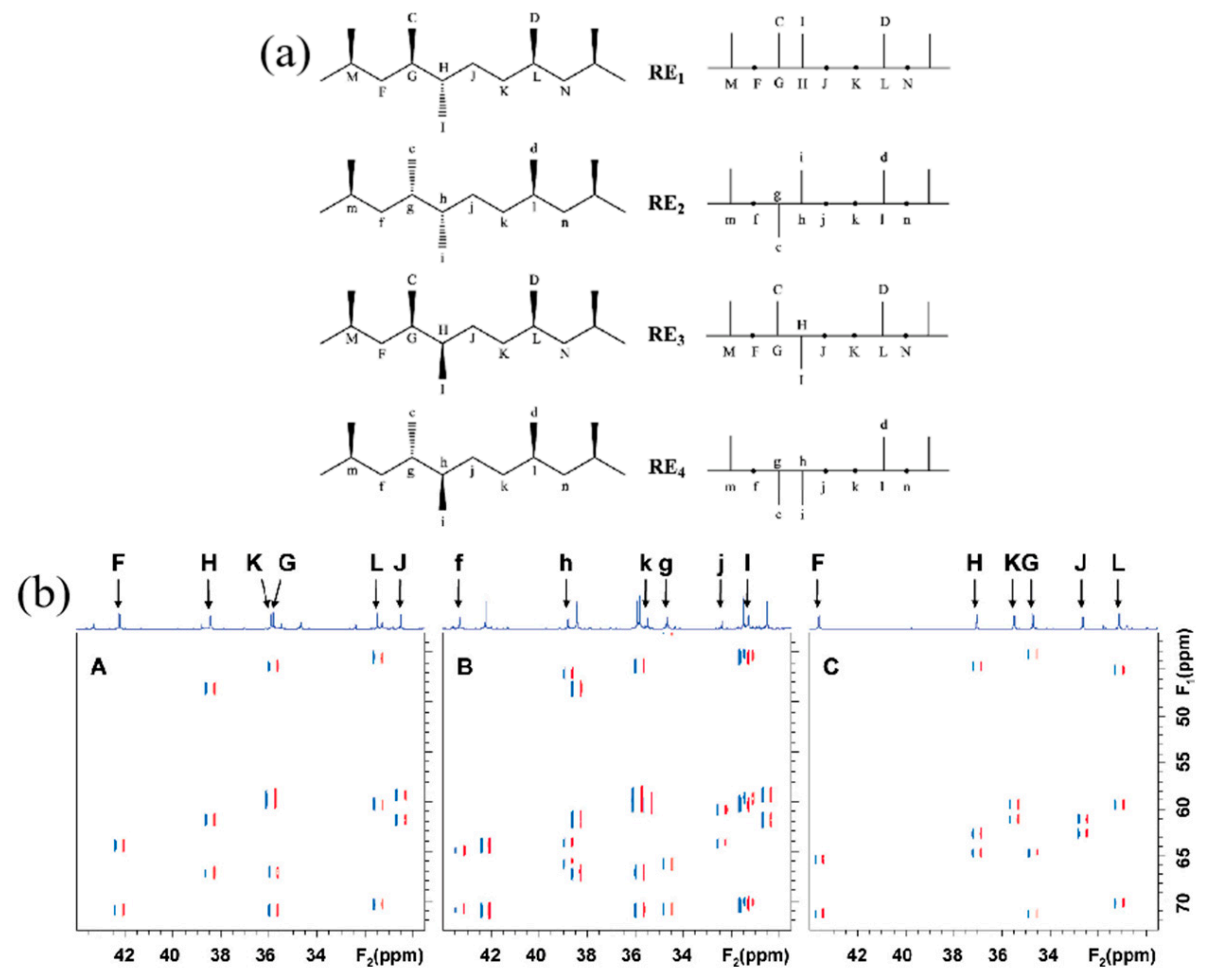
(**a**) 3D structures and Fisher projections of 2,1-insertion regioerrors in iPP; (**b**) fragments of 2D INADEQUATE. (**A**): 2,1-insertion regioerrors (RE_1_) in the iPP. (**B**): 2,1-insertion regioerrors of the iPP; only resonances for the regioerrors with lower concentration (RE_2_) are labeled. (**C**): 2,1-insertion regioerrors (RE_3_) in the iPP. NMR time was 2–3 days. Reproduced from Ref. [105] with permission from the American Chemical Society.

**Figure 4:**
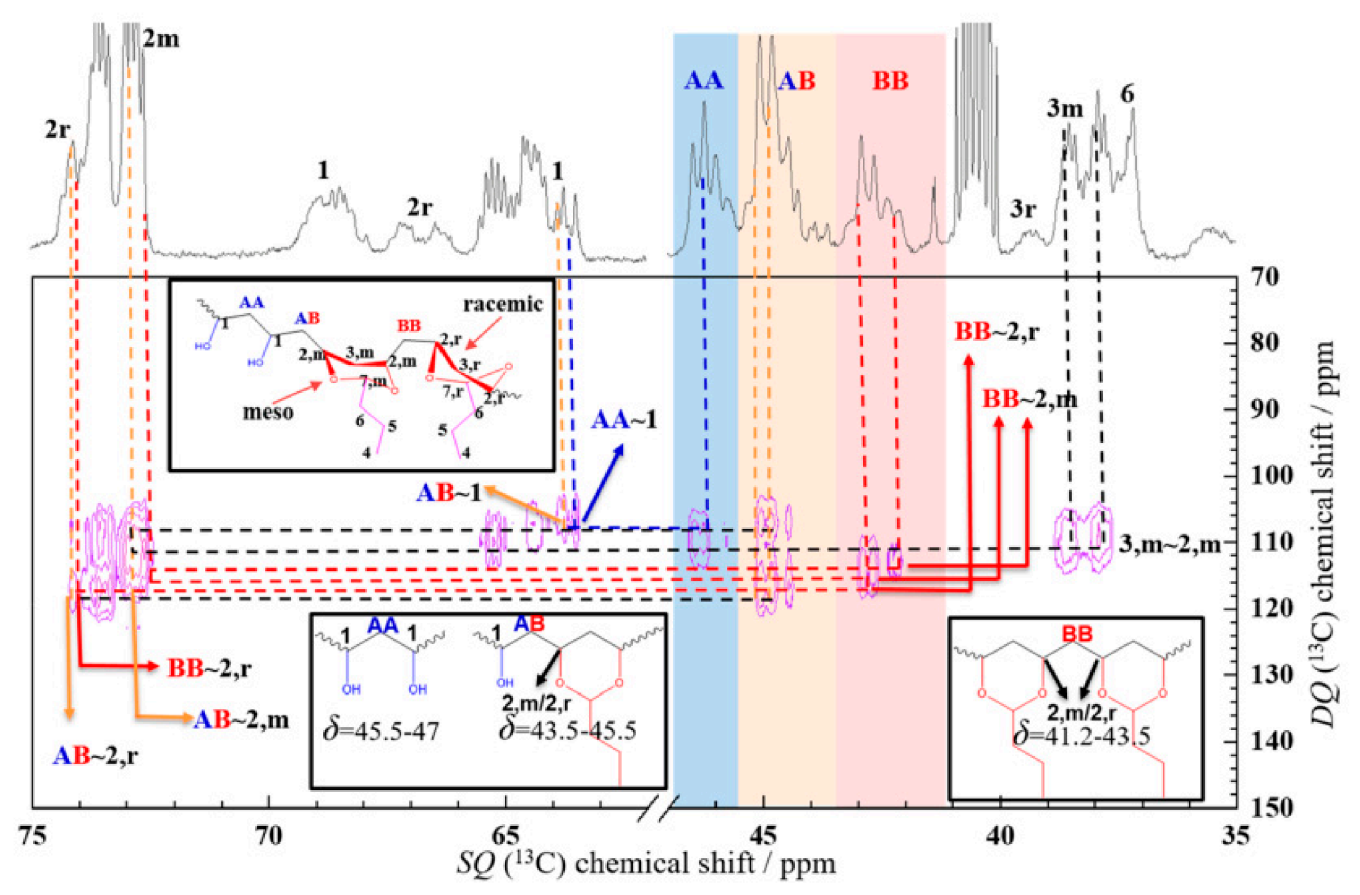
2D INADEQUATE of 10% ^13^C-enriched PVB. Reproduced from Ref. [107] with permission from the American Chemical Society.

1. ^13^C-enriched sodium carboxymethyl cellulose (CMC) and cellulose acetate (CA). J_CC_ = 61 Hz was used. For CMC with a substitution degree of 0.91, the 2D INADEQUATE revealed four distinct anhydroglucose units (AGUs), including unsubstituted, 2-mono-, 3-mono-, and 6-monosubstituted units, evidenced by multiple C1-C2 spin pairs detected at different DQ frequencies (three at 189 ppm and one at 181 ppm). Similarly, for CA with a substitution degree of 2.54, the technique identified four different AGUs: 2,3,6-tri-, 2,3-di-, 2,6-di-, and 3,6-disubstituted units. However, the technique showed limitations in the 70–80 ppm spectral region where signal overlap made precise assignment of C2, C3, and C5 resonances challenging [89].
2. Ethylene–Propylene copolymer. Revealed that methylene carbons at 29.93 ppm (δδ+), 30.31 ppm (γδ+), 27.37 ppm (βδ+), and 37.45 ppm (αδ+) formed a chain connected to a methine carbon at 33.13 ppm. Additional assignments included methyl groups at 20.59 ppm (αα methylene-connected) and 19.90 ppm, linked through a methine at 30.74 ppm, supporting configurational insights for propylene diads [90].
3. Atactic poly(vinyl alcohol). Provided insights into connections between methine and methylene carbons. For instance, methine 9 exhibited four distinct cross-peaks (9-a, 9-b, 9-g, and 9-g”), and methine 10 showed only one cross-peak (10-g”). By analyzing these connectivities and referencing previous assignments based on pentad and hexad configurations, specific carbon environments were identified. Methine 9 was linked to mrrr (meso-racemic-racemic-racemic) and methine 10 to mrrm. Similarly, methylene carbons a, b, and c were linked to mrrrm, mrrrr, and rrrrr configurations, respectively [91].
4. Ethylene–Propylene copolymer. Pentad-level assignments of methine carbon resonances in a stereoregular Ethylene–Propylene copolymer were reported by revealing connectivities between methyl and methine carbons. The experiment was done on a 270 MHz NMR with 0.4 g/cm^3^ polymer. Both sensitivity and resolution are low [92].
5. Polypropylene. The 2D INADEQUATE demonstrated the connectivity between methyl and methine carbons as well as between methine and methylene carbons. Using this approach, the researchers were able to connect nine methine carbon resonances to their corresponding methyl pentad resonances, enabling assignment of most methine peaks to specific stereosequences. The analysis confirmed three heptad resonances (mmmmmm, mmmmmr, and rmmmmr) and five of the six expected pentad resonances (mmmr, rmmr, mmrr, rmrr, and mmrm), with the mrmr/rrrr/rrrm/mrrm sequences appearing as a single peak [93].
6. Atactic poly(1-pentene) and poly(1-hexene). 2D INADEQUATE on a 270 MHz NMR was used to experimentally validate previous ^13^C NMR tacticity assignments. The method revealed ^13^C–^13^C connectivities, such as those between C3–C4 and C3–C2 carbons, which supported triad and partial pentad tacticity assignments for the polymer chains. For instance, splitting patterns in the C3 and C4 regions indicated the presence of triad (rr, mr, mm) and pentad-level microstructures, aligning with theoretical chemical-shift predictions. Additionally, higher-field peaks in the methylene carbon region of the backbone, previously predicted but unobserved in isotactic samples, were confirmed. The resolution and sensitivity of the study are low, and the results need to be reexamined with high-field NMR [94].
7. Stereoregular polybutene–propylene. The 2D INADEQUATE on a 270 MHz was employed. By tracing connectivities between directly bonded carbons (e.g., methyl–methine and methine–methylene pairs), the validity of triad (e.g., PPP, BPP, BPB) and tetrad (e.g., BPPP, BPPB) sequence assignments was established. For instance, peak C in the methyl carbon region was assigned to the PPP triad due to its connectivity with methylene carbons central to PP diads. Similarly, tetrad sequences like BPPP and BPPB were resolved through cross-peaks linking methyl, methine, and methylene carbons. The technique also clarified overlapping resonances, confirming prior assignments of BP-centered tetrads and B-centered triads. These results aligned with both reaction probability model predictions and chemical shift calculations based on the γ-effect method [95].
8. Regioirregular polypropylene. Containing up to 40 mol% inverted propylene units. 2D INADEQUATE on a 400 MHz NMR was used. Identified four distinct sequences incorporating H-H and T-T units, including isolated H-H/T-T units, single inverted units (1-r) within H-T sequences, two successive inverted units, and sequences with consecutive inversions. By mapping carbon connectivities, the 2D INADEQUATE spectra confirmed the exclusive presence of a meso H-H unit in sequences with isolated inverted units [96].
9. Cyclopolymer derived from ethyl α-[(allyloxy)methyl]acrylate. The 2D INADEQUATE on a 400 MHz NMR was employed to determine the ring size and structure, confirming formation of five-membered rings in the polymer backbone. By correlating γ-gauche shielding effects (e.g., a 6.8 ppm shift difference for methylene groups adjacent to quaternary carbons), the cis and trans isomers were differentiated. The 2D INADEQUATE data conclusively demonstrated that intramolecular cyclization occurred with high fidelity, ruling out competing six-membered ring formation or mixed ring sizes [97].
10. Regioirregular poly(1-butene). 2D INADEQUATE on a 270 MHz was used to assign the peaks corresponding to carbons in H-T, isolated H-H, T-T, and sequences with 1-r, 2-r, and successive (2U-r) inversions. For instance, the ^13^C–^13^C correlations revealed distinct chemical shifts for carbons in these sequences, such as peaks at 28.5, 33.2, and 43.2 ppm for the 1-r sequence. The technique also clarified unresolved peaks in earlier studies, providing detailed assignment of regioirregular poly(1-butene) by distinguishing overlapping signals from CH, CH_2_, and CH_3_ groups and mapping them to specific structural motifs [98].
11. Poly(vinyl chloride). Resolved contradictions in prior ^13^C NMR assignments. By analyzing the connectivities between methylene and methine carbons, 2D INADEQUATE revealed two distinct mmr tetrad peaks, one of which overlapped with the mrm peak. This clarified discrepancies between observed and Bernoullian-predicted relative peak areas for methylene tetrads. Additionally, pentad and hexad sequence assignments were achieved by correlating centered methine carbons in pentads with methylene tetrads. The results validated the Bernoullian propagation model for Poly(vinyl chloride) polymerization, confirming the statistical distribution of stereosequences. The method enabled precise differentiation of overlapping peaks and shoulders in the 1D spectrum, demonstrating the power of 2D INADEQUATE for resolving complex polymer microstructures. However, due to low resolution and low sensitivity of the experiments, some of the assignments may need to be reexamined [99].
12. The 2D INADEQUATE NMR analysis of poly(α-^13^C-styrene) revealed key structural insights into the termination mechanisms of styrene free radical polymerization. Cross-peaks in the spectrum were assigned to specific C-C couplings, including H-H and H-T enchainments. Notably, resonances in region C confirmed the presence of α-carbon (Cα) linkages, such as Cα–Cα bonds, which are indicative of termination via radical combination. Comparisons between low- and high-molecular-weight polymers showed that Cα–Cα signals diminished in higher-molecular-weight samples, consistent with termination-derived structures rather than random H-H propagation. The technique selectively enhanced ^13^C–^13^C spin pairs formed during termination, providing direct evidence for combination as the dominant termination pathway. However, due to low resolution and low sensitivity of the experiments, some of the assignments may need to be reexamined [100].
13. Polyisobutene oligomers. For BF_3_-catalyzed polybutene, the 2D INADEQUATE confirmed the presence of a tert-butyl group at one chain end and a methylvinylidene double bond at the other, with six resolved carbon connectivities from both ends. The quaternary carbons (δ 38.0 ppm) were linked to methylene (δ 59.4 ppm) and methyl groups (δ 31.2 ppm), validating the expected structure. In contrast, AlCl_3_-catalyzed polybutene revealed an isopropyl group (methyl δ 25.77 ppm, methine δ 24.06 ppm) at one end and a trisubstituted double bond at the other, with seven connectivities mapped from the double bond and six from the isopropyl terminus. This technique resolved ambiguities in prior studies, disproving assumptions of T-T isobutylene addition or carbocation rearrangements and highlighting the complexity of AlCl_3_-catalyzed polymerization mechanisms. Coupling constants (e.g., sp^3^-sp^2^ ≈ 42 Hz, sp^2^-sp^2^ ≈ 70 Hz) further supported structural assignments [101].
14. Luteophanol A. A ^13^C-enriched sample was used; it identified key correlations between quaternary carbons (C-22 and C-34) and their neighboring carbons. These connectivities included C-22/C-21, C-22/C-23, C-22/C-59, as well as C-34/C-33, C-34/C-35, and C-34/C-60. This data provided definitive evidence for linking the three partial structures (a–c) into the complete molecular framework, resolving ambiguities in the assembly of the polyhydroxylated C57-linear chain. The 2D INADEQUATE results were pivotal in establishing the positions of methyl branches and the exo-methylene group, thereby validating the overall planar structure [102].
15. Polypropylene. Assigned the methine resonances, complementing their 1D spectral analysis. The 2D INADEQUATE was recorded with a partly tactic low-molecular-mass polypropylene oil with predominant m diads on a 600 MHz NMR and at 70 °C, which provided a CH-CH_3_ correlation map. From this correlation map, the authors deduced the sequence of methine peaks from low to high field as mmmmmm, mmmmmr, rmmmmr, mmmr, and mmrr. Notably, they observed that the main component of the rmmr pentad overlapped with the broad multiplet containing other mr- and rr-centered pentads. This 2D approach yielded 12 certain assignments that significantly simplified the analysis of 1D spectra for predominantly isotactic and syndiotactic samples. The experiment required substantial time (9 days) due to the low natural abundance of ^13^C, using 40 mg of sample dissolved in tetrachloroethane-d_2_ [103].
16. Cyclopolymers. Derived from N-substituted-N-allyl-2-(methoxycarbonyl)allylamines. Revealed the dominant repeating unit consists of a five-membered ring structure. Cross-peaks corresponding to AX or AB spin systems were observed, with the stronger intensity signals aligning along a diagonal line in the F_1_ and F_2_ frequency domains, confirming the five-membered ring skeleton. The 2D INADEQUATE also supported the assignment of trans and cis configurations for the methylene carbons attached to the ring, with the trans isomer being the major component [104].
17. Isotactic propylene (iPP). A significant improvement in sensitivity for 2D INADEQUATE was achieved with the development of a high-temperature (120–130 °C), 10 mm, 400 MHz, ^13^C-optimized cryoprobe (Figure 3). The large NMR tube diameter is particularly advantageous for polymer NMR studies, as it allows for an increased sample volume within the probe’s active region. Equally important is the probe’s capability to operate at elevated temperatures, which is essential for certain polymer characterizations. Overall, a 5.5-fold increase in sensitivity was observed with this cryoprobe at 125 °C compared to a conventional 400 MHz broadband 10 mm probe. The performance of the cryoprobe was demonstrated through the study of the 2,1-inverse insertion in isotactic propylene. The 2D INADEQUATE unambiguously showed carbon connectivities and stereochemistries of isolated 2,1-regioerrors (RE_1_–RE_4_). Resolved low-concentration regioerrors (e.g., RE_2_ at 0.4 mol%) that were previously challenging to characterize. Revealed distinct carbon networks for RE_1_, RE_2_, and RE_3_, including reversed chemical shift sequences for RE_2_ compared to RE_1_. Key assignments included methyl group resonances influenced by “gamma gauche” effects, enabling differentiation of threo and erythro configurations. The analysis corrected prior misassignments (e.g., RE_2_) and confirmed RE_3_ with additional C-N assignments. This work provided the first definitive experimental characterization of all carbons associated with these regioerrors, critical for understanding catalyst mechanisms and polymer properties [105].
18. Poly(propylene-co-1-octene) (E/O). The 2D INADEQUATE enabled by the high-temperature 10 mm cryoprobe was used to unambiguously assign 2,1-insertion regioerrors in a E/O. Despite the low octene content (4 mol%) and the rarity of regioerrors (0.55 mol%), the method resolved C-C connectivities in both the octene side chains and the regioirregular propylene sequences. This provided detailed insights into the microstructure, including the stereochemical identity of the regioerrors (assigned as RE_3_ via HOESY experiments), which are critical for understanding polymerization catalyst mechanisms and their impact on polymer properties. Cryoprobe reduced the experimental time from an estimated 2.6 months (with conventional probes) to just 2.6 days, making such analyses feasible [106].
19. Poly(vinyl butyral) (PVB). A 10% ^13^C-enriched PVB sample was synthesized to enhance sensitivity and achieve an adequate S/N. The 2D INADEQUATE (Figure 4) revealed distinct ^1^J_CC_ couplings between backbone carbon atoms, allowing differentiation of diad sequences: AA (vinyl alcohol–vinyl alcohol), AB (vinyl alcohol–vinyl butyral), and BB (vinyl butyral–vinyl butyral). Specifically, the methylene carbon resonance at δ(^13^C) = 45.1 ppm showed coupling with both δ(^13^C) = 63.8 ppm and 72.9 ppm, confirming its assignment to the AB sequence, while the peak at δ(^13^C) = 46.2 ppm coupled only with δ(^13^C) = 63.5 ppm, identifying it as part of the AA sequence. No ^1^J_CC_ correlation was observed for the peak at δ(^13^C) = 37.2 ppm, confirming its origin from a side-chain methylene group rather than the polymer backbone. Furthermore, BB sequence carbons exhibited couplings exclusively with methine carbons assigned to vinyl butyral units (#2,m or #2,r). This direct C-C correlation provided critical evidence for the precise assignment of all resolved ^13^C NMR signals and enabled accurate characterization of the sequence distribution in PVB (Figure 4) [107].

### 2.3. Mixture Examples

This subsection highlights applications in which 2D INADEQUATE was deployed to resolve carbon connectivity in multi-component systems, demonstrating that direct ^13^C–^13^C bond mapping can distinguish spectroscopically overlapping species and assign connectivities without the need for chromatographic separation.

1. M-cresol novolak dimers. Resolved overlapping signals in complex mixtures, enabling the identification of symmetric and dissymmetric isomers (I–V). Isomer VI remained partially unassigned due to spectral overlap [108].
2. 2-alkylnaphthalenes. The spectrum of 2-tert-butylnaphthalene was clarified, avoiding complications from the AB effect observed in DQ spectra of doubly labeled molecules. Confirmed the signal sequences for aromatic carbons and validated consistency across varying concentrations and solvents (e.g., comparison with dilute solutions and acetone data). It was critical for resolving ambiguities in alkyl-substituted naphthalene systems, particularly for sterically hindered tert-butyl derivatives, where non-bonded interactions caused significant deshielding effects [109].
3. Trans- and cis-myrtanol. Established unambiguous assignments. For instance, C-10 (δ 66.25 ppm) exhibited a correlation peak with the methine carbon at δ 38.12 ppm (C-2), which in turn correlated with a methine carbon (δ 42.87 ppm, C-1) and a methylene carbon (δ 18.79 ppm, C-3). This direct through-bond connectivity mapping enabled the complete and self-consistent structural assignment of the bicyclic framework, resolving ambiguities in prior studies [110].
4. Cellobiose and maltose, quinoline and 3-acetyl pyridine, n-propanol and 1,2-propanediol. Enabled the detection of 22 separate signals for cellobiose and 23 for maltose, corresponding to their alpha and beta anomeric forms. 2D INADEQUATE revealed three distinct carbon connectivity chains in each disaccharide: one from the common pyranosyl group and two from the individual anomeric forms, allowing unambiguous assignment of all carbon signals to specific nuclei. The technique also measured C-1/C-2 coupling constants, revealing approximately a 10 Hz difference between alpha and beta orientations of the free hydroxyl group in the pyranose ring, while showing that C-1/C-2 coupling in the pyranosyl sugar was virtually independent of glycosyl linkage conformation. For mixture analysis, INADEQUATE proved particularly valuable in distinguishing compounds that would otherwise be confused based on chemical shifts alone. In a mixture of quinoline and 3-acetyl pyridine, it demonstrated that the “pyridine-like” resonances (123.5, 135.7, and 149.8 ppm) did not connect together, ruling out actual pyridine presence. Similarly, for a mixture of n-propanol and 1,2-propanediol, it showed that signals at 25.8 and 67.8 ppm (which resemble tetrahydrofuran) were not connected as they would be in tetrahydrofuran, proving they originated from different compounds. The technique’s unique ability to map direct C-C connectivities makes it powerful for mixture analysis where conventional chemical shift recognition fails due to signal overlap [111].

### 2.4. Miscellaneous Examples

This subsection collects applications of 2D INADEQUATE to non-traditional systems—including gas-phase molecules, surface-bound species, and isotopically enriched biomaterials—illustrating the technique’s adaptability beyond conventional solution-state analysis.

**Figure 5:**
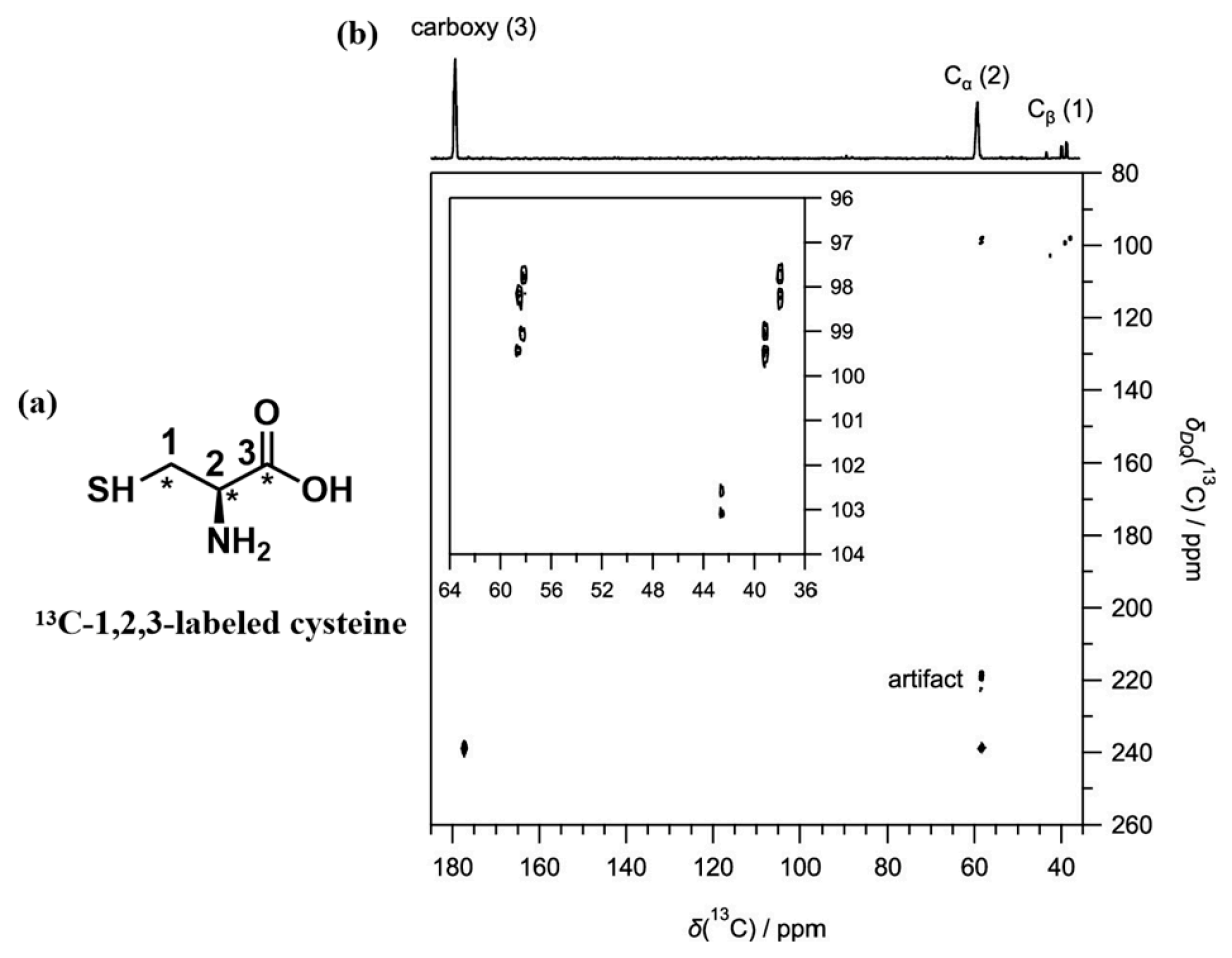
(**a**) ^13^C-1,2,3-labeled cysteine. * ^13^C-labeled. (**b**) ^13^C–^13^C INADEQUATE NMR spectrum of ultrasmall gold nanoparticles, functionalized with ^13^C-1,2,3-labeled cysteine (pH 12). Reproduced from Ref. [114] with permission from the American Chemical Society.

1. Propene gas at natural isotopic abundance. The spectrum was acquired for 14 h at 30 °C, with the sample at 0.51 M concentration dictated by its vapor pressure (1.5 × 10^6^ Pa). Despite the low concentration, rapid ^13^C spin-lattice relaxation (T_1_ values of 0.53 s, 0.43 s, and 2.4 s for carbons CH_2_, CH and CH_3_) enabled efficient signal averaging using a 2 s recycle delay. This highlights the advantage of gas-phase NMR for experiments requiring repetitive signal acquisition, as shorter relaxation times compared to solution-phase systems reduce total measurement time. While 2D INADEQUATE is less critical for routine gas mixture analysis, the work underscores its feasibility for structural elucidation in volatile systems, particularly when combined with cryoprobes to enhance sensitivity [112].
2. Perhydro-as-indacene frameworks, ^1^H-cyclopent[c]indene derivatives, dodecahydroacenaphthylenes, decahydro-^1^H-cyclopent[*d*]indene, 2,8-tetramethylenebicyclo [3.3.0]-octane, octahydro-^2^H-1,4a-ethanonaphthalene, and the tetracyclic bishomohypostrophane. The carbon connectivity was obtained, combined with computational NMR shift predictions, and enabled definitive structural assignments for these previously unknown or uncharacterized compounds, revealing unexpected complexity in a reaction thought to be thoroughly understood after decades of industrial application [113].
3. Cysteine molecules bound to gold nanoparticles. The analysis revealed that the terminal carboxy carbon at 179 ppm couples with the neighboring C_α_ carbon at 60 ppm (sum of chemical shifts: 239 ppm). Three distinct species of C_β_ were observed coupling with their respective C_α_ carbons, evidenced by signals at 39 ppm/59 ppm (sum: 98 ppm) and 40 ppm/59 ppm (sum: 99 ppm). Although the coupling between C_β_ at 43 ppm and its corresponding C_α_ at 60 ppm (expected sum: 103 ppm) could not be detected due to low S/N, the 2D INADEQUATE conclusively demonstrated the connectivity between different C_β_ carbons and their respective C_α_ carbons, as well as their coupling to carbonyl carbons. This provided definitive evidence for the structural integrity of cysteine molecules on the gold nanoparticle surface, confirming that the three observed C_β_ signals correspond to three different chemical environments of cysteine bound to the nanoparticle rather than decomposition products or impurities (Figure 5) [114].
4. Mouse fed with ^13^C-labeled amino acids. From the liver sample analysis, Python-based INADEQUATE Network Analysis (PyINETA, version 2.0) constructed 67 INADEQUATE networks, with the majority (52 out of 67) containing just two peaks, while 15 networks were longer (up to 8 peaks maximum in Network 8). Database matching revealed that 46 of these networks corresponded to known compounds, enabling the identification of 21 specific metabolites, including amino acids (alanine, glutamine, leucine, lysine, threonine, glutamic acid, isoleucine, valine, and proline), organic acids (lactic acid and chloroacetic acid), amino sugars, amino alcohols, amino sulfonic acids, and various amines. Demonstrated how INADEQUATE could detect carbon connectivity patterns even when networks were fragmented—for example, lactic acid (a 3-carbon compound) appeared as two separate horizontal networks (Networks 7 and 55) due to a missing vertical connection. The study successfully tracked the tissue-specific distribution of these ^13^C-labeled metabolites across 9 different mouse tissues, revealing 3 distinct patterns: 13 compounds enriched specifically in the liver (Type-A), 2 compounds depleted in the liver but enriched in other tissues (Type-B), and 4 compounds enriched in both the liver and other tissues (Type-C). Importantly, the researchers also tracked 16 unknown compounds that did not match database entries, such as Uk-16, which showed exclusive enrichment in liver tissue, demonstrating 2D INADEQUATE’s value for investigating novel metabolites [115].

## 3. Improved 2D INADEQUATE

**Figure 6:**
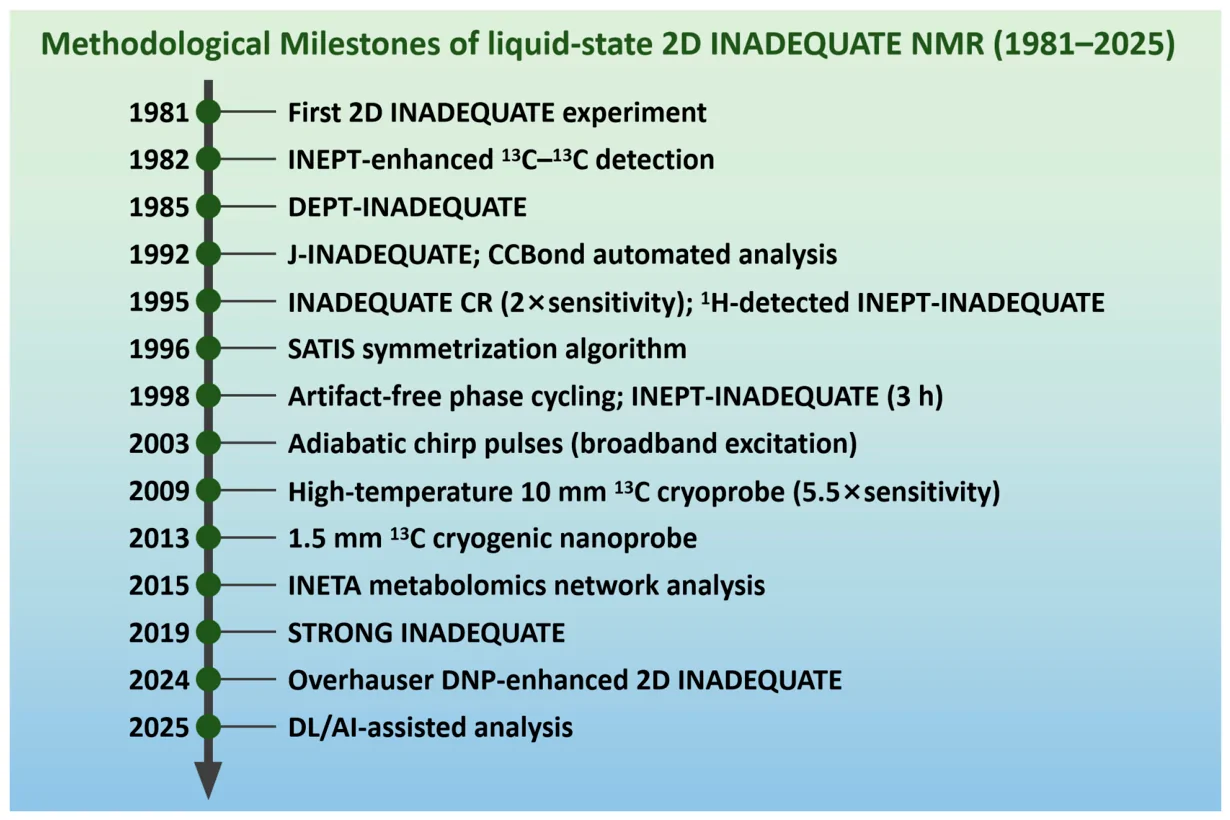
Chronological development of liquid-state 2D INADEQUATE methodology, 1981–2025. 1981: first 2D INADEQUATE experiment [1,2]; 1982: INEPT-enhanced ^13^C–^13^C detection [116]; 1985: DEPT-INADEQUATE proposed [117]; 1992: J-INADEQUATE [118] and CCBond automated analysis [119,120,121]; 1995: INADEQUATE CR with ~2-fold sensitivity gain [122,123] and ^1^H-detected INEPT-INADEQUATE at natural abundance [124]; 1996: SATIS symmetry-based processing [125]; 1998: artifact-free phase cycling [126] and 3 h INEPT-INADEQUATE with O_2_ pressurization [127]; 2003: broadband excitation via adiabatic chirp pulses [128]; 2009: high-temperature 10 mm ^13^C cryoprobe with 5.5-fold sensitivity gain [105,106]; 2013: 1.5 mm ^13^C-optimized cryogenic nanoprobe [47]; 2015: INETA network analysis for metabolomics [129]; 2019: STRONG INADEQUATE for small J_CC_ couplings [130]; 2024: Overhauser DNP-enhanced 2D INADEQUATE [131]; 2024–2025: DL/AI-assisted spectral denoising, reconstruction, and automated analysis [115,132,133,134,135,136,137,138,139,140,141,142,143,144].

The chronological development of some methodological milestones is summarized in Figure 6, which spans from the first 2D INADEQUATE experiment in 1981 to the most recent AI-assisted spectral analysis. These developments can be grouped into three overlapping phases: (i) pulse-sequence innovations: INEPT/DEPT enhancement, J-compensation, CR, adiabatic pulses, and DNP; (ii) hardware-driven sensitivity gains: 5 mm and 10 mm cryoprobes, and the 1.5 mm cryogenic nanoprobe; and (iii) data-processing and automated-analysis tools: CCBond, FRED, SATIS, INETA, and Deep Learning (DL). Having outlined this historical trajectory, we now compare the individual developments quantitatively. Before examining each methodological improvement in detail, Table 2 provides a consolidated comparison of all sensitivity-enhancement strategies discussed in Section 3, Section 4 and Section 5, summarizing the sensitivity gains and key limitations of each approach. This comparison is intended to help practitioners select the most appropriate variant for their specific experimental constraints.

**Figure 7:**
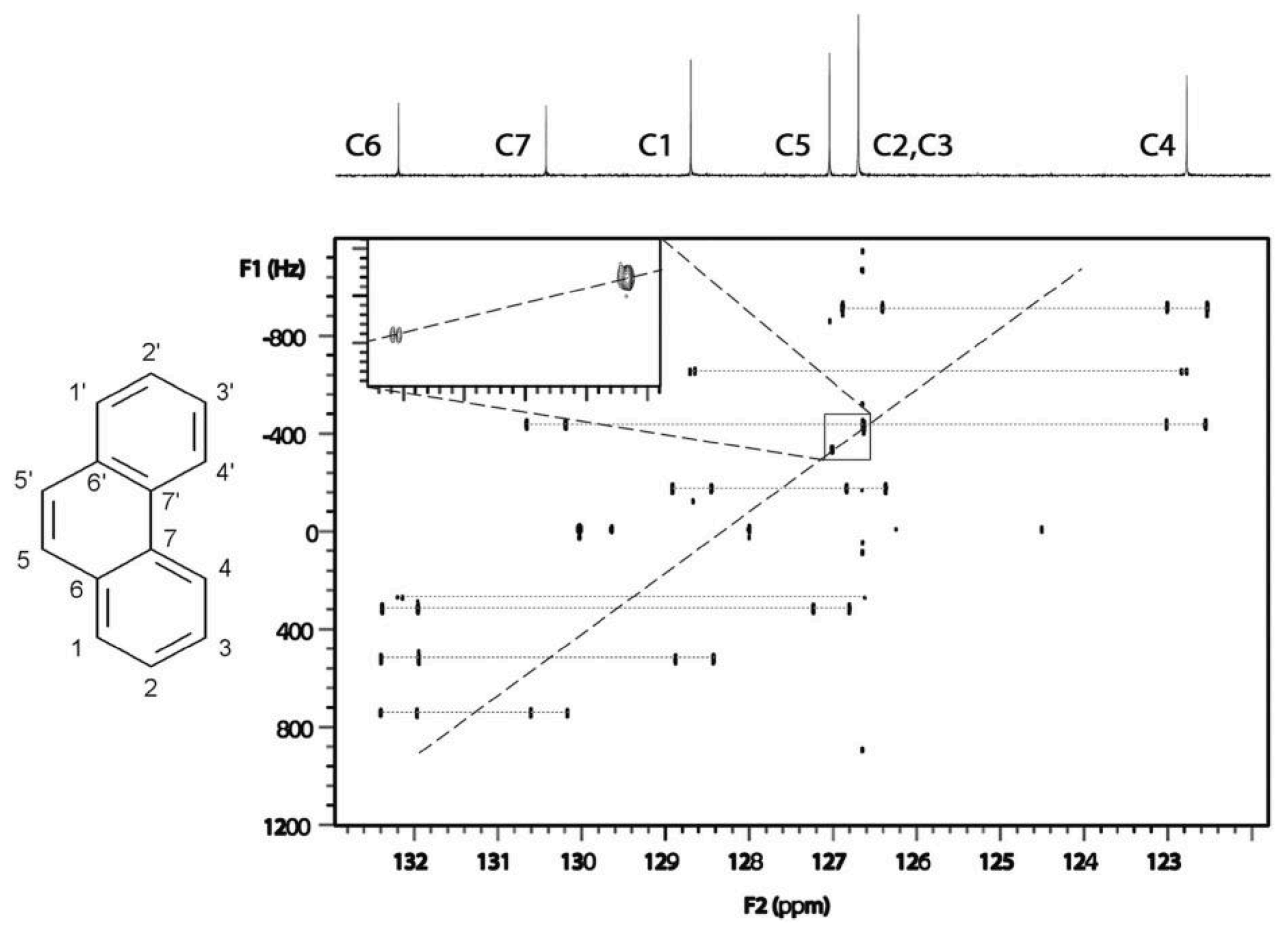
A refocused 2D STRONG INADEQUATE applied to the phenanthrene molecule using a fixed length of polarization transfer delay equal to the refocusing delay τ (0.01 s). Reproduced from Ref. [130] with permission from John Wiley & Sons.

**Figure 8:**
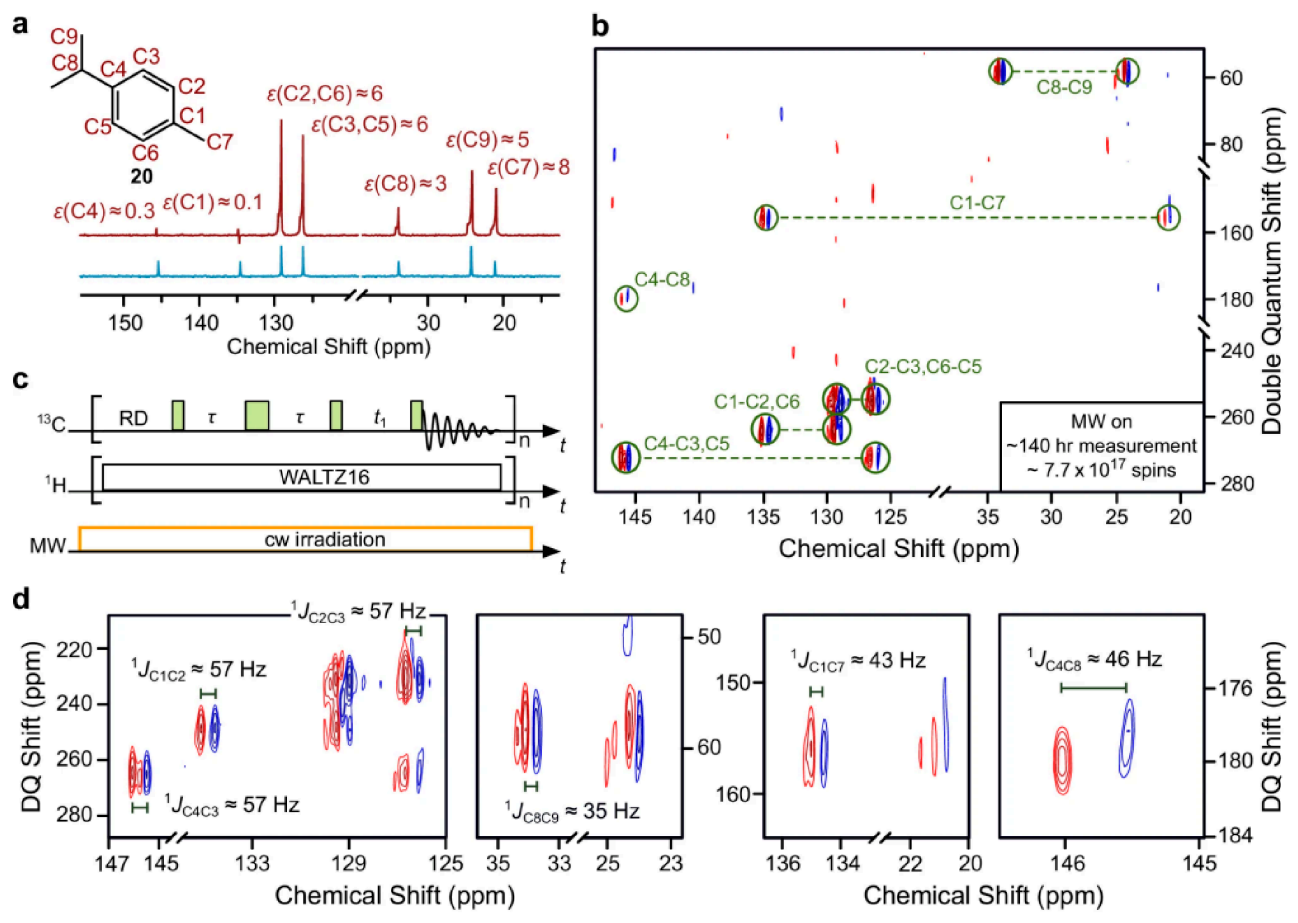
(**a**) The structure and 1D ^13^C NMR spectrum of p-cymene under DNP (red) and Boltzmann (blue). (**b**) DNP-enhanced ^13^C–^13^C NMR INADEQUATE of ~17mg p-cymene (Nat. ab.). Peaks (marked by green circles) corresponding to correlated nuclei (green labels) are connected by green dashed bars. (**c**) Pulse sequence for DNP-enhanced 2D INADEQUATE. (**d**) Expanded views of (**b**), showing measurements of J_CC_. Reproduced from Ref. [131] with permission from Springer Nature.

1. The J-compensated 2D INADEQUATE (J-INADEQUATE) NMR technique enhances sensitivity and robustness in detecting C-C connectivities by addressing mismatches between the delay time (τ = 1/2J) and actual homonuclear spin–spin coupling constants (J). This method employs a J-compensated spin-echo sequence derived from an operator analogy between RF pulses and homonuclear coupling evolution, enabling efficient conversion of in-phase magnetization into antiphase/double-quantum coherence across a wide range of J values. By integrating a tailored spin-echo element, J-INADEQUATE mitigates signal loss caused by J-τ mismatches. Experimental validation on crotonaldehyde demonstrated significant intensity gains (e.g., ~60% increase for C-5/C-6 doublets) and improved resolution for spin pairs with extreme J values (e.g., C-2/C-3 with J = 67 Hz and C-1/C-4 with J = 7.4 Hz), which are poorly resolved in conventional INADEQUATE. This approach broadens the applicability of 2D INADEQUATE to complex molecules with heterogeneous couplings or suboptimal τ selection, offering reliable structural elucidation where standard methods fail [118]. It was reported that a typical range of J_CC_ is 30–80 Hz and the average is 55 Hz [153]. There is a review article that highlights how the J_CC_ coupling constant varies significantly depending on bond type and substituents. Overall, J_CC_ values span a very broad range—from less than 0.5 Hz to as high as 230.4 Hz. However, for typical organic compounds without extreme or unusual substituents, characteristic ranges can be identified: single bonds (Csp^3^–Csp^3^) generally exhibit J_CC_ values of approximately 35–65 Hz; double bonds (Csp^2^–Csp^2^) fall in the range of about 50–80 Hz; and triple bonds (Csp–Csp) show much larger couplings, typically between 130 and 180 Hz. For most common organic molecules under standard conditions, the J_CC_ values usually lie within the range of approximately 40–70 Hz [154].
2. 2D INADEQUATE is prone to rapid-pulsing artifacts arising from incomplete longitudinal relaxation between scans. These artifacts manifest as spurious multiple-quantum (MQ, Δm = 0, ±2) and single-quantum (SQ, Δm = ±1) signals, degrading spectral quality and complicating data interpretation. Bourdonneau and Ancian addressed this issue by introducing a novel phase-cycling scheme that compensates for imperfections in excitation pulses (e.g., 90° and 180° pulses) and suppresses artifacts through paired-scan acquisition. By alternating pulse phases and receiver phases across consecutive scans, residual magnetization from noncoupled ^13^C nuclei is effectively canceled, eliminating MQ/SQ artifacts while preserving genuine double-quantum coherence signals. Experimental validation on menthol in CDCl_3_ demonstrated significant improvements in spectral clarity and sensitivity, enabling faster data collection without compromising resolution. The method outperforms traditional approaches, such as pulsed field gradients (PFGs) or composite pulses, by integrating error compensation directly into phase cycling, making it particularly valuable for time-intensive 2D-INADEQUATE experiments [126].
3. Nielsen et al. enhanced sensitivity by introducing CR pulses and PFGs to suppress artifacts and concentrate signal intensity into a single doublet line of the ^13^C–^13^C cross-peaks. This approach, termed 2D INADEQUATE CR, achieves a theoretical sensitivity gain of ~2.18-fold over conventional 2D INADEQUATE. Experimental validation on menthol demonstrated improved S/N while maintaining selectivity for specific ^13^C–^13^C couplings. Additionally, merging left and right doublet spectra after frequency shifts recovers full sensitivity, producing a homonuclear decoupled-like spectrum. The method preserves robustness against miscalibrated delays and avoids missing quaternary carbon connectivities, which are problematic in ^1^H-detected variants [122]. By employing a systematic strategy for designing optimal coherence transfer experiments, Nielsen et al. further detailed their INADEQUATE CR method, which doubles the sensitivity compared to the original INADEQUATE approach. This improvement is achieved through selective rotations and nonselective pulse sequences that efficiently convert DQC to SQC, minimizing signal loss. Experimental validation on [^13^C_2_]L-alanine demonstrated superior multiplet and coherence order selectivity, with peak intensities approximately twice those of conventional INADEQUATE experiments. The method also maintains compatibility with PFGs, ensuring robust suppression of unwanted signals without compromising sensitivity [123].
4. The modified INADEQUATE CR variant improves sensitivity by selectively transferring DQC to only one line of each doublet, doubling the signal intensity compared to conventional INADEQUATE. Off-resonance effects (ORE), which degrade performance in large spectral widths (e.g., camphor spanning ~200 ppm), are mitigated using Levitt’s global pulse sequence compensation, preserving the doubled sensitivity. Experiments on camphor demonstrated a theoretical 2.18-fold enhancement in sensitivity for INADEQUATE CR over conventional INADEQUATE, with minimal cross-talk between spectral lines [145,146]. This holds true exclusively when transversal relaxation processes are not taken into account.
5. The 2D INADEQUATE CR is remarkable for its robustness to J-coupling mismatches, with even a severe 40% deviation between actual and nominal J values yielding 86.02% signal in the desired peak while maintaining only 2.95% signal in the unwanted neighboring doublet line. This tolerance ensures substantial sensitivity advantages remain intact even with imperfect parameter matching. Additionally, by leveraging symmetry properties of the four INADEQUATE CR variants, researchers can generate pure 2D absorption spectra and “merged” spectra that provide apparent homonuclear decoupling, greatly enhancing spectral interpretation and making the technique particularly valuable for analyzing molecules with diverse J-coupling constants [147].
6. Conventional ^13^C rectangular pulses with a maximum B_1_ (γB_1_max) of 20 kHz (corresponding to 12.5 µs and 25 µs for 90° and 180° pulses, respectively) induce significant ORE across the broad 200 ppm chemical shift range typical for ^13^C nuclei. These off-resonance artifacts can severely attenuate or entirely suppress signals in multi-pulse 2D INADEQUATE experiments, which rely on precise coherence transfer. To mitigate this limitation, practical implementations of 2D INADEQUATE pulse sequences must incorporate strategies to compensate for such effects. Critical to this are the 180° inversion and refocusing pulses, which are particularly susceptible to bandwidth limitations. The integration of adiabatic pulses—which provide broadband excitation and robustness against resonance offsets—can dramatically improve sensitivity by ensuring efficient coherence manipulation across the entire spectral window [128].
7. The STRONG INADEQUATE technique, a modified version of the conventional 2D INADEQUATE NMR experiment, was developed specifically for detecting small C-C coupling constants between equivalent carbon atoms in symmetrical molecules where traditional methods fail (Figure 7). Unlike standard INADEQUATE approaches that suffer from signal collapse in symmetrical systems, this method utilizes one-bond C-H coupling to create effective chemical shift differences between otherwise equivalent nuclei. The technique produces significantly simplified spectral patterns, particularly for ^n^J_CC_ couplings where n ≥ 3, yielding a clean antiphase doublet with ^n^J_CC_ separation in the F_1_ dimension that remains uncomplicated even when the symmetrical HC···C’H’ system couples to other protons in the molecule. Experimental validation demonstrated its effectiveness in measuring ^3^J_CC_ couplings in challenging systems, with benzene yielding a value of 10.1 Hz and phenanthrene showing a ^3^J(C4, C4′) coupling of 4.7 Hz. The method overcomes critical limitations of previous techniques like nondecoupled INADEQUATE, 2QHMBC, and SYMONA, which produce complex, highly split patterns that cause peak cancellation for small couplings (<10 Hz), thereby enabling reliable detection of C-C couplings in strongly coupled systems where conventional approaches are ineffective [130].
8. Overhauser DNP-enhanced 2D ^13^C–^13^C INADEQUATE was designed to overcome the low sensitivity of conventional thermal-equilibrium INADEQUATE (Figure 8). Unlike dissolution DNP and PHIP (Parahydrogen-Induced Hyperpolarization), which generate transient magnetization and cannot sustain prolonged F_1_ incremental acquisition, this 9.4 T liquid NMR platform achieved steady-state hyperpolarization through 263 GHz continuous microwave irradiation of polarizing agents. The polarization is transferred from a selected source nucleus to its scalar-coupled partners, visualizing unreactive quaternary and carbonyl carbons in cross-peaks. A complete stable DNP-enhanced 2D INADEQUATE was collected over 140 h with only ~17 mg of natural-abundance p-cymene. Although the 140 h NMR time remains substantial, the critical advantage of steady-state Overhauser DNP over dissolution DNP or PHIP is that the hyperpolarization is maintained continuously throughout the entire F_1_ increment series, enabling true 2D acquisition. Dissolution DNP, by contrast, provides only a single transient enhancement that decays within seconds, precluding conventional 2D acquisition. The enhancements of the cross-peaks correspond to the average 1D enhancements of the two involved carbons, allowing precise quantification of all ^1^J_CC_ and full assignment of the molecular carbon connectivities. Severe paramagnetic relaxation stemming from radicals restricts the direct application of this liquid DNP strategy to macromolecules; this bottleneck may be alleviated by adopting spatially isolated polarizing agents. Furthermore, additional improvement of sensitivity could include a combination of DNP with cryo-probe technologies and ^1^H detection of ^13^C resonances, which substantially shortens NMR time while retaining the benefits of hyperpolarization gain [131].

## 4. 2D INEPT-INADEQUATE

Polarization transfer from protons effectively benefits coupled pairs of carbon atoms when both are protonated and, to a lesser degree, when only one is protonated. The polarization approach offers distinct advantages over the NOE method. First, it provides higher signal enhancement, making it more effective for amplifying detectable signals. Second, the polarization approach allows for the use of a relaxation agent to shorten ^1^H T_1_, which accelerates data acquisition and improves experimental efficiency. In contrast, the NOE is diminished when a relaxation agent is applied, as the agent disrupts the NOE mechanism that relies on specific spin-spin interactions. This key difference highlights the polarization approach’s superiority in scenarios requiring both strong signal amplification and optimized relaxation times. The DEPT-INADEQUATE technique was proposed, but its structural utility comes at the cost of increased experimental complexity [117]. Its reliance on extensive phase cycling to suppress artifacts significantly prolongs the total experimental time compared to simpler INEPT protocols. Furthermore, DEPT-INADEQUATE is more susceptible to hardware imperfections, such as pulse phase miscalibration, field-frequency instability, and probe tuning, which can reduce transfer efficiency by 50% or more if not meticulously optimized. It also demands longer presaturation sequences to randomize spin phases, further extending experiment duration. While DEPT-INADEQUATE achieves 80–90% of theoretical transfer efficiency under ideal conditions, mismatches in coupling constants (e.g., J_CC_ or J_CH_) can lead to suboptimal results, unlike INEPT’s potentially greater robustness. Thus, here, we focus on INEPT-INADEQUATE [117].

1. A significant gain is expected if the experiment starts from ^1^H magnetization and detects ^13^C magnetization. Combining INEPT (Insensitive Nuclei Enhanced by Polarization Transfer) with the INADEQUATE sequence enhanced sensitivity by transferring proton polarization to ^13^C nuclei, achieving a ~2.6-fold improvement in S/N compared to conventional nuclear Overhauser techniques. This allowed efficient detection of double-quantum coherence transitions in pyridine, demonstrating the utility of the method for elucidating molecular frameworks even with limited sample quantities. The pulse sequence and NMR spectra shown were 1D, although the possibility of converting to 2D was mentioned [116].
2. 2D INADEQUATE spectrum of an 80% methyl salicylate sample (pressurized with O_2_ at 3 atm and cooled to 15 °C) was acquired in 3 h, revealing all seven correlations clearly. Combining 2D INEPT-INADEQUATE further enhanced sensitivity for protonated-quaternary carbon pairs, achieving a sixfold time saving compared to conventional methods [127].
3. Weigelt and Otting introduced a ^1^H-detected INEPT-INADEQUATE, which has much better sensitivity than the conventional ^13^C-detected INADEQUATE technique by utilizing ^1^H magnetization both as the starting point and for detection. The authors successfully addressed the major challenge of suppressing ^12^C-bound proton signals by more than a factor of 10,000 through the implementation of PFGs combined with a four-step phase cycle to select the desired coherence-transfer pathway, enabling detection of the much weaker signals from protons bound to ^13^C–^13^C fragments. Experimental validation using a 2M sucrose solution demonstrated the technique’s ability to trace carbon chains through H-C-C-H fragments without additional ^13^C shift information when all carbons carry protons, while achieving an approximately 13-fold improvement in S/N compared to conventional INADEQUATE, making carbon connectivity studies more feasible for biological molecules where sample quantity is often limited [124].
4. A study utilized ^1^H-detected 2D INEPT-INADEQUATE to resolve structural ambiguities in circumdatin A. This technique enabled the unambiguous mapping of C-C connectivities in the zwitterionic benzodiazepine scaffold. Specifically, the INEPT- INADEQUATE experiment (optimized for ^1^J_CC_ = 55 Hz) established direct one-bond connections such as C3–C4, C4–C5, C12–C13, and C15–C16 in circumdatin A [155].

In both the ^13^C-detected 2D INEPT-INADEQUATE and ^1^H-detected 2D INEPT-INADEQUATE techniques, singlets are generated in the F_1_ dimension. For the ^13^C-detected 2D INEPT-INADEQUATE, the F_2_ dimension exhibits antiphase doublets, which arise from C-C couplings. These doublets directly reflect the presence of scalar interactions between adjacent carbon nuclei. In the ^1^H-detected 2D INEPT-INADEQUATE method, the proton–proton couplings are often sufficiently small that they do not resolve into distinct peaks, leading to all signal intensity converging into a single peak in the F_2_ dimension when couplings remain unresolved. Notably, while the ^1^H-detected approach offers higher sensitivity, it lacks adequate resolution for some applications—such as the structural analysis of saturated carbons in polyolefins—where all protons are resonated in about a 1 ppm chemical shift range in the F_2_ dimension. The theoretical sensitivity enhancement of ^1^H-detected 2D INEPT-INADEQUATE compared to ^13^C-detected 2D INEPT-INADEQUATE is approximately (γ_1H_/γ_13C_)^1^.^5^ ~ 8. However, in practice, such a significant sensitivity gain is rarely achieved with ^1^H detection, primarily due to the complexity of ^1^H multiplets. In contrast, ^13^C detection typically yields signals with natural linewidths of just a few hertz, whereas ^1^H detection often results in broad, unresolved proton multiplets with much wider line widths. ^13^C-detected 2D INEPT-INADEQUATE also typically uses a 10 mm probe, in which the ^1^H coil is positioned externally. This outer-coil configuration reduces ^1^H sensitivity due to increased distance from the sample. When all factors are considered, the ^13^C-detected 2D INEPT-INADEQUATE with a 10 mm probe retains some advantages for certain applications. It is important to note that pairs of quaternary carbons, commonly present in organic compounds, are not detectable by polarization transfer methods that depend on one-bond J_CH_ coupling.

Limitations and When Alternatives Are Preferable: Despite the substantial methodological advances summarized in Table 2, a balanced assessment requires acknowledgment of the persistent limitations that constrain the broader adoption of 2D INADEQUATE. First, the fundamental sensitivity barrier remains formidable. Second, practical NMR time spans a wide and often prohibitive range—from a couple of hours for the exceptionally favorable case [127], through 36–35 h for moderate-sized natural products at natural abundance [27,30], to 9 days for polypropylene methine-region analysis [103], and an estimated 2.6 months for poly(propylene-co-1-octene) regioerrors on conventional probes [105,106]. Even with the 5.5-fold gain of the high-temperature 10 mm cryoprobe, the experiment still required 2.6 days [106]. Third, sample-mass requirements are substantial: natural-abundance experiments typically demand ≥5–10 mg on a cryoprobe-equipped spectrometer (e.g., 10.4 mg for dehydrocrebanine [58]; 70 mg for the cyclobuta[b]pyrimidodiindole study [84]), and 40 mg or greater for polymer analyses without isotopic enrichment [103]. Although specialized probes (e.g., the 1.5 mm cryoprobe) have reduced this to ~1 mg for small molecules [47], such hardware remains uncommon. Fourth, for many routine structural problems, HSQC, HMBC, or LR-HSQMBC remain the more practical and informative choice: for proton-rich molecules with well-resolved 1H spectra, for quality-control or high-throughput workflows where spectrometer time is at a premium, or when only C-H connectivity (rather than direct C-C connectivity) is required. In such cases, the superior sensitivity and shorter NMR time of these heteronuclear experiments render INADEQUATE unnecessary. Finally, it must be emphasized that INEPT-based variants (both 13C-detected and 1H-detected INEPT-INADEQUATE) are inherently unable to detect quaternary–quaternary C-C pairs, which are often the very connectivities most difficult to establish by any other means. Consequently, the choice between conventional INADEQUATE and INEPT-INADEQUATE must be guided by the specific structural question at hand. These limitations do not diminish the unique and irreplaceable role of 2D INADEQUATE when absolute certainty in C-C connectivity is required; rather, they define the boundaries within which the technique delivers its greatest value.

## 5. Computerized ^13^C–^13^C 2D INADEQUATE Analysis

**Figure 9:**
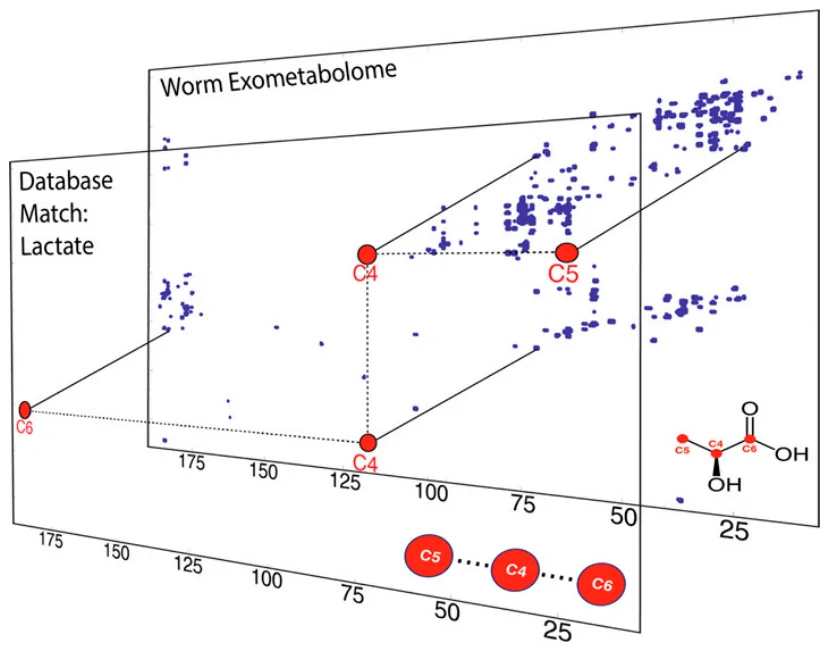
A semi-automated approach, INETA, for untargeted analysis of INADEQUATE datasets using an in silico INADEQUATE database. Reproduced from Ref. [129] with permission from the American Chemical Society.

1. The 2D INADEQUATE NMR experiment, optimized for sp^3^-sp^3^ C-C couplings (J_CC_ = 40 Hz), enabled unambiguous identification of the carbon backbone of bistramide A (bistratene A). Using an automated analysis program (CCBond), the spectral data were interpreted through a parametric model involving Lorentzian and sine line-shape convolutions, combined with response surface mapping and nonlinear regression. This approach resolved all C-C bonds, except two sp^2^-sp^2^ bonds (C2–C3 and C36–C37) [119].
2. Advancements in automated 2D INADEQUATE analysis were described, using a parametric model and response surface mapping, implemented in the CCBond program. These improvements allow reliable bond detection in spectra with root-mean-square S/N as low as 2.5, enabling analysis of samples containing as little as 20 μmol of compound. Key enhancements include modeling all four quadrants of the hypercomplex spectrum, addressing truncation effects, and eliminating manual phasing through phase-sensitive parameterization. The method resolves overlapping bond patterns via three-dimensional parameter space exploration and leverages prior knowledge to reduce computational complexity, extending the practical applicability of 2D INADEQUATE for structural elucidation [120].
3. The 2D INADEQUATE NMR technique was employed to determine C-C bond connectivity in low-concentration samples of bistramide A and cholesterol. For bistramide A (71 μmol), the method enabled the detection of 35 C-C bonds. In the case of cholesterol (20 μmol), despite limited spectral resolution and low S/N, the automated analysis successfully identified 34 bond signals, although some ambiguities arose due to overlapping transitions and second-order effects. The program CCBond was instrumental in extracting bond information from noisy data, significantly improving the sensitivity compared to manual interpretation. Limitations such as undetectable bonds between carbons with nearly identical chemical shifts or severe second-order effects were noted, but these could be addressed with complementary data or higher-resolution acquisition [121].
4. Provided absolute confirmation of 35 out of 53 possible C–C bonds in Saponin PT-2 through ^13^C–^13^C connectivities. Due to 2D IADEQUATE’s low sensitivity, computerized spectral analysis with the CCBond3 program was essential to identify weak signals that could not be resolved visually. The analysis revealed that methyl and quaternary carbon bonds were preferentially detected, while methylene groups were less observable due to shorter relaxation times and broader spectral lines. Additionally, the pulse sequence optimized for single-bond scalar couplings failed to detect C–C double bonds. Despite these limitations, the experiment resolved ambiguities in sugar peak assignments and confirmed spatial relationships under strong solvent resonances [149].
5. The 2D INADEQUATE NMR technique, combined with the CCBond software and a high-sensitivity carbon nanoprobe, enabled the unambiguous assignment of 20 out of 22 C-C bonds in obscurinervidine using only 26 mg of sample. Despite the inherently low sensitivity of INADEQUATE experiments, the nanoprobe and advanced data analysis overcame challenges such as spectral noise and under-digitization, allowing bond detection with >99.5% certainty. The two undetected bonds (C13–C18 and C17–C18) were resolved by default through structural context. This approach significantly reduced material requirements and the NMR time compared to conventional methods, which typically demand hundreds of milligrams and extensive measurement periods [150].
6. Despite challenges posed by low sensitivity due to limited sample quantity (33 mg) and a low S/N, the technique enabled the identification of key C-C bonds. Four of the five C-C bonds in the α-L-rhamnopyranoside sugar moiety (C2′–C3′, C3′–C4′, C4′–C5′, and C5′–C6′) were unequivocally detected, while the C2′–C3′ bond was resolved using the computational program CCBond to overcome second-order effects. In the aglycone chromone core, the experiment revealed four direct C-C bonds (C6–C5, C5–C10, C10–C4, and C4–C3) and three additional bonds (C9–C8, C8–C7, and C7–C6) through visual analysis and computational assistance [151].
7. The study utilized 2D INADEQUATE NMR spectroscopy in conjunction with a high-sensitivity nanoprobe and FRED software to determine the carbon skeleton of 3-hydroxypregnane-2,16-dione. This approach allowed direct detection of C-C bonds. Using just 11 mg of sample (30 μmol), the complete carbon structure was elucidated in 62 h on a 500 MHz spectrometer. The nanoprobe enhanced sensitivity, while FRED automated data analysis, generating a fully assigned carbon framework within 1.5 h. This marked a successful application of both technologies to an unknown compound, achieving structural resolution with ~10-fold less material than conventional INADEQUATE requirements [152].
8. Symmetrization procedures replace each pair of symmetry-related points a, b with the smaller of the two values, giving a higher S/N as spurious noise peaks are suppressed. The study introduced symmetry-based processing methods (S1 and S2) to exploit the antisymmetry of cross-peaks relative to SQ and DQ diagonals, while correcting for sotopic shift-induced asymmetry. The S/N ratio improved 2-fold, equivalent to reducing experimental time by a factor of 4 [148].
9. A symmetry-based data-processing algorithm called Symmetrization Algorithm for Treatment of 2D INADEQUATE Spectra (SATIS) was introduced. This approach leverages the inherent symmetry of the four-line AX/AB subspectra in 2D INADEQUATE data, combining intensities from all four lines to enhance sensitivity and reduce false positives/negatives. Applied to a natural-abundance ^13^C spectrum of cholesterol at 100 MHz, SATIS improved clarity, enabling the identification of 22 correlations, including challenging quaternary-methyl carbon couplings. However, correlations involving long relaxation times or degenerate chemical shifts remained ambiguous. The method also mitigated baseline noise and avoided artifacts from incomplete subspectra, though weak signals buried in noise were still difficult to resolve [125].
10. Isotopically labeled caenorhabditis elegans were studied using a 1.5 mm ^13^C-optimized cryoprobe. Spectra of 99% ^13^C-labeled histidine (at 2 mM concentration) yielded significantly higher S/N in just 4 h, compared to 48 h required for natural-abundance histidine (200 mM) with much lower S/N. The labeled samples also revealed additional correlations beyond standard DQ couplings, attributed to multiple interacting spin systems. These artifacts were managed using 2D INADEQUATE rules (e.g., DQ frequency = sum of single quantum shifts), which allowed discrimination of directly bonded ^13^C pairs while retaining utility for fragment connectivity across heteroatoms. A custom software, INETA, semi-automatically identified 53 and 49 carbon networks in endo- and exometabolomes, respectively, matching 31 compounds to a database of ^13^C spectra. The approach demonstrated the feasibility of untargeted metabolomics using ^13^C NMR, particularly for identifying metabolites in complex mixtures without chromatographic separation, and highlighted metabolic shifts under heat shock conditions, such as increased lactate and succinate levels (Figure 9) [129].

## 6. Future Perspectives

The preceding sections have documented how methodological, hardware, and computational advances have progressively lowered the barriers to applying 2D INADEQUATE. In this section, we turn to the developments most likely to shape its next chapter. Four converging trajectories stand out: the rapid maturation of AI tools for spectral processing and interpretation; the deepening integration of experimental INADEQUATE data with quantum-chemical computation; the continued evolution of NMR hardware across the full field-strength spectrum, from ultra-high-field cryoprobes down to benchtop permanent-magnet systems; and the as-yet untapped potential of NUS to compress experiment times dramatically. Each of these avenues addresses a distinct bottleneck—sensitivity, throughput, interpretive expertise, or structural validation—and together, they sketch a path toward 2D INADEQUATE becoming faster, more automated, and more broadly accessible than at any point in its nearly five-decade history. We discuss these prospects in turn below.

1. AI in 2D INADEQUATE: DL is rapidly transforming NMR spectroscopy, and several of these advances are directly transferable to 2D INADEQUATE. Transformer-based models that elucidate structures from multidimensional NMR (NMRMind) [132] and automated peak-assignment frameworks for 1D spectra (NMRformer) [138] point toward fully automated interpretation of 2D INADEQUATE data. Scalable deep-learning reconstruction has been demonstrated for accelerated multidimensional NMR [134,135], including accelerated pure-shift spectroscopy [133,136] and Laplace NMR reconstruction [137,142]; applied to 2D INADEQUATE, such reconstruction would permit substantial undersampling of the F_1_ dimension. Deep neural networks such as DN-Unet improve the S/N of liquid-state NMR spectra [143], and robust chemometrics-based denoising has been extended to multidimensional data [144]; for 2D INADEQUATE, denoising is arguably the most immediately impactful application, because the cross-peaks buried near the noise floor are precisely those most often missed in manual interpretation. DL has likewise been applied to resolution enhancement [139] and to cross-modal retrieval between ^13^C spectra and structures for compound identification [140], with a recent review summarizing the state of the art [141]. We anticipate that the combination of AI-assisted denoising, automated peak picking, and network-analysis tools such as INETA/PyINETA [115,129] will convert 2D INADEQUATE interpretation from a manual, expert-driven task into a routine computational workflow.

2. Integration with Computational Chemistry: Combining experimental ^1^J_CC_ values with quantum-chemical calculations adds a powerful validation dimension to INADEQUATE-based elucidation. For the hydrogen-poor depsidone perisalazinic acid, experimental 2D INADEQUATE-derived ^1^J_CC_ values were compared against DFT-computed values to evaluate 81 candidate structures and identify the best-fit structure [56]. Similarly, INADEQUATE-derived connectivities combined with computational NMR shift predictions enabled definitive assignments of complex perhydro-as-indacene frameworks and related industrial byproducts [113]. Looking forward, systematic DFT calculation of ^1^J_CC_ values—and, eventually, machine-learned shift and coupling predictors—should allow INADEQUATE data to serve not only for connectivity mapping but also for stereochemical and electronic-structure validation.

3. Emerging Hardware and the Low-Field (Benchtop): Higher field strengths, improved cryoprobes—including the high-temperature 10 mm ^13^C probe [105,106] and the 1.5 mm cryogenic nanoprobe [47]—and microcoil designs will continue to improve sensitivity and reduce sample requirements. An important question raised during review is whether INADEQUATE can be deployed on benchtop/low-field (<100 MHz) instruments, whose use is growing for reaction profiling and structural analysis. At present, the answer is largely negative for natural-abundance 2D work: (i) the already low ^13^C sensitivity is further reduced at lower fields; (ii) ^13^C chemical-shift dispersion in Hz scales linearly with B_0_, so spectral crowding in both dimensions becomes severe and near-degenerate pairs proliferate; and (iii) cryoprobe technology is generally unavailable at benchtop field strengths. For ^13^C-enriched samples or small molecules with a large quantity and well-dispersed spectra, 2D INADEQUATE-type experiments at low field may become feasible as permanent-magnet and digital-receiver technology improves, and compact hyperpolarization accessories could further extend feasibility.

4. Non-Uniform Sampling (NUS): NUS, now widely available on modern spectrometers, reduces the effective NMR time of 2D experiments by 50–75% by undersampling the F_1_ dimension and reconstructing the full spectrum via compressed-sensing or maximum-entropy algorithms. Although, to our knowledge, no NUS 2D INADEQUATE paper on solution-state NMR has yet appeared, the technique is directly applicable and—combined with cryoprobe or DNP enhancement and AI-based reconstruction—represents an obvious but currently unexploited opportunity.

## 7. Practical Considerations

We consolidate and expand the practical guidance of this review into the following organized recommendations:

(i) Sample and concentration. Both ^1^H and ^13^C pulses must be calibrated using the sample solution. The minimum concentration should allow acquisition of a ^13^C spectrum with S/N > 3 via a single scan with one 90° pulse.

(ii) Relaxation management. Measure the T_1_ relaxation times of isolated ^13^C nuclei before the experiment; if necessary, shorten them to ~2–3 s using Cr(acac)_3_ (typically 0.012–0.030 M), which is particularly effective for quaternary carbons. Caution: polar functional groups (-OH, -NH_2_) may complex Cr(acac)_3_, causing severe line broadening or signal loss; in conventional INADEQUATE, relaxation agents may also suppress the NOE. For INEPT-based versions, measure the ^1^H T_1_ values instead.

(iii) Repetition time. A repetition time of 1.26 × T_1_ maximizes signal per unit time (versus 5 × T_1_ for full relaxation). Because two carbon signals contribute, use the shorter T_1_ of ^13^C for ^13^C-detected experiments, or of ^1^H for INEPT-enhanced versions [156]. Rapid-pulsing artifacts can additionally be eliminated by the paired-scan phase-cycling scheme of Bourdonneau and Ancian [126].

(iv) Choice of J_CC_. Initiate with J = 40 Hz; increasing to 65 Hz may recover weak or missing connectivities. Extending the delays τ around the 180° pulse enhances sensitivity to long-range (^2^J_CC_, ^3^J_CC_) connectivities, whereas shortening τ favors unusually large ^1^J_CC_ values (e.g., acetylenic carbons) [156]. For strongly coupled pairs, changing solvent (e.g., CDCl_3,_ C_6_D_6_) and adjusting τ from 1/(4J_CC_) to 3/(4J_CC_) may recover missing cross-peaks [52].

(v) Pulses. The hard-pulse condition often fails for ^13^C at high field: a 15 µs 90° pulse corresponds to γB_1_ ≈ 16.7 kHz, whereas the ^13^C window at 600 MHz spans 30 kHz. Refocusing 180° pulses are especially vulnerable; adiabatic ^13^C 180° pulses ensure uniform inversion across the full bandwidth [128].

(vi) Read pulse. Contrary to common practice (e.g., Bruker inadph/inadphsp sequences, which employ a 90° read pulse), the optimal read-pulse angle for 2D INADEQUATE could be 120° [156], as this maximizes transfer from DQC to observable SQC.

(vii) Digital resolution. High digital resolution in the F_2_ dimension is essential to resolve true ^13^C–^13^C doublets and distinguish them from residual SQ singlets; acquire a large number of t_2_ points and compensate with minimal interscan delays.

Failure modes and troubleshooting: The most common failure modes encountered in the surveyed literature are: (a) strong coupling and near-degenerate shifts (e.g., C-11/C-12 in anthracimycin [52]; C-4′/C-5′ in ascorbigen A [55]), addressed by solvent change, τ adjustment, or higher field; (b) long quaternary-carbon T_1_, addressed by relaxation agents or INEPT variants; (c) off-resonance attenuation at high field, addressed by adiabatic pulses; (d) invisibility of quaternary–quaternary pairs in INEPT-based variants, addressed by conventional or CR INADEQUATE; and (e) rapid-pulsing artifacts, addressed by phase cycling [126] or longer recycle delays. Table 3 summarizes representative experimental parameters (sample mass, field, probe, NMR time) drawn from the studies surveyed in this review, providing a quick planning reference for practitioners.

**Figure 10:**
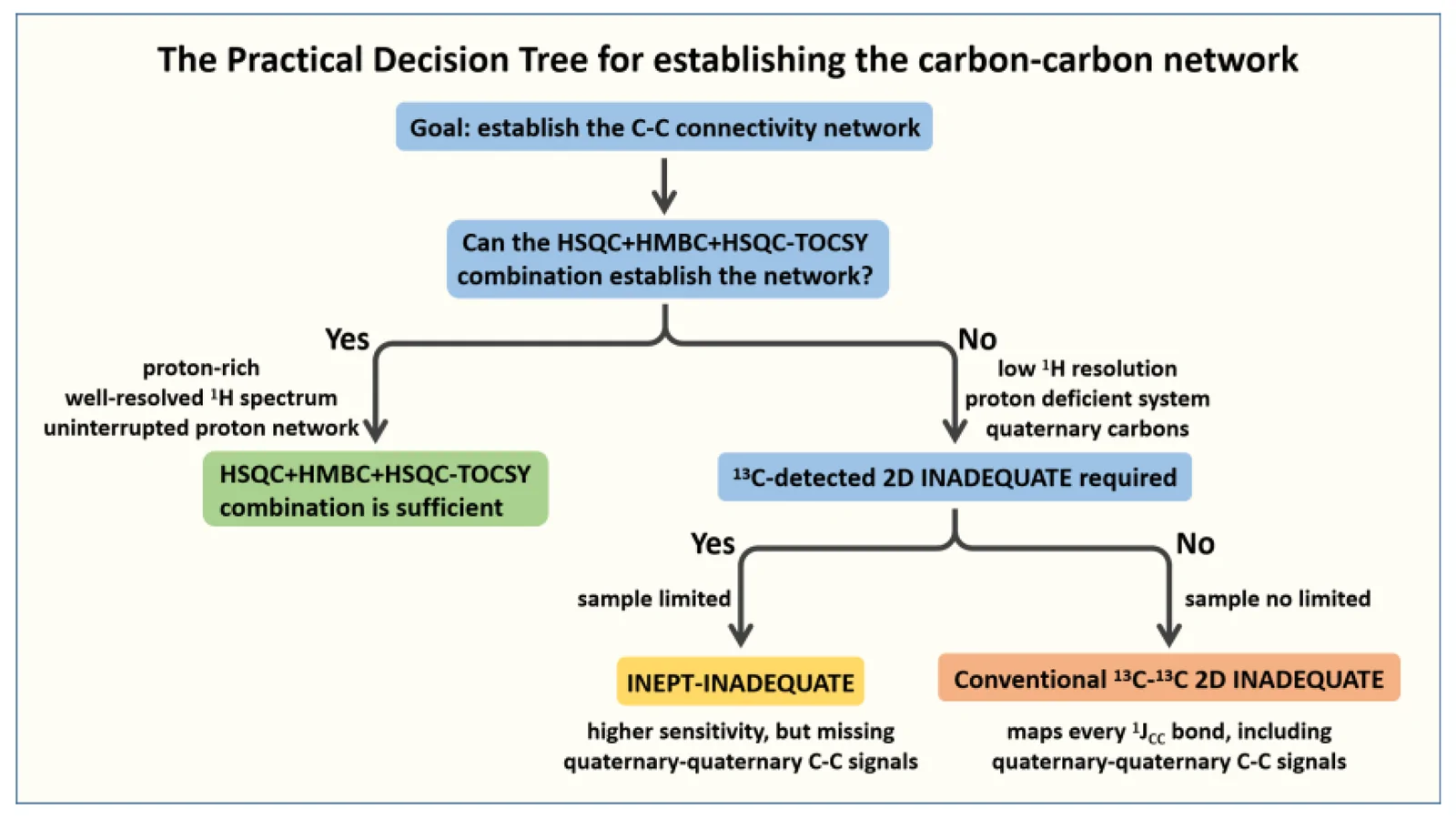
Practical decision tree for establishing the C-C network. For proton-rich molecules with well-resolved ^1^H spectra and uninterrupted proton networks, the HSQC + HMBC + HSQC-TOCSY combination is sufficient. When low ^1^H resolution, proton deficiency, or quaternary carbons preclude this, a ^13^C-detected 2D INADEQUATE is required: INEPT-INADEQUATE if the sample is limited (higher sensitivity, but missing quaternary–quaternary connectivities), or conventional ^13^C–^13^C 2D INADEQUATE if the sample is not limited (the only method mapping every J_CC_ bond, including quaternary–quaternary pairs).

To translate the foregoing discussion into a form that can be applied in everyday practice, Figure 10 provides a simple decision tree whose explicit goal is to establish the complete C-C connectivity network. The tree first asks whether the combination of HSQC, HMBC, and HSQC-TOCSY can establish the network: for proton-rich molecules with well-resolved ^1^H spectra and an uninterrupted proton relay, the answer is yes, and this fast, sensitive combination is sufficient. When, however, the ^1^H resolution is low, the system is proton-deficient, or quaternary carbons interrupt the proton network, the heteronuclear combination fails, and a ^13^C-detected 2D INADEQUATE experiment becomes required. The tree then branches on sample quantity. If the sample is limited, INEPT-INADEQUATE offers substantially higher sensitivity at the cost of missing quaternary–quaternary C-C connectivities because the polarization transfer relies on one-bond J_CH_ couplings. If the sample is not limited, conventional ^13^C–^13^C 2D INADEQUATE is the method of choice, as it is the only experiment that directly and unambiguously maps every J_CC_ bond, including quaternary–quaternary pairs. Finally, we note that ^1^H-detected INEPT-INADEQUATE, although it may deliver higher S/N, faces the same issues as the HSQC + HMBC + HSQC-TOCSY combination—namely, reliance on limited ^1^H resolution—and the authors of this review do not have hands-on experience with this variant; it is therefore not summarized in this decision tree, and readers are encouraged to explore it themselves.

## 8. Summary

Liquid-state ^13^C–^13^C 2D INADEQUATE NMR spectroscopy has proven to be an indispensable technique for unambiguous C-C connectivity mapping despite its inherent sensitivity challenges. Over four decades since its introduction, this method has consistently provided definitive structural evidence in cases where conventional NMR approaches fail, particularly for molecules containing quaternary carbons, fully substituted aromatic systems, and complex polymer architectures. The review demonstrates that 2D INADEQUATE has successfully resolved longstanding structural ambiguities across diverse chemical domains—from natural products to sophisticated polymer microstructures.

Nevertheless, a critical assessment reveals that the technique’s adoption has been limited by practical barriers: natural-abundance experiments typically require ≥ 5–10 mg of sample and hours to weeks of spectrometer time, restricting its use to well-resourced laboratories. Strong-coupling artifacts remain a persistent challenge, and INEPT-based variants cannot detect quaternary–quaternary pairs. These limitations must be weighed against the unique and irreplaceable information that 2D INADEQUATE provides.

The integration of cryogenic probe technology exemplifies how hardware innovations have made previously impractical analyses feasible. Complementary developments—including specialized pulse sequences like 2D INEPT-INADEQUATE and computerized analysis algorithm-assisted interpretation—have further expanded the technique’s utility. Modern implementations have transformed ^13^C–^13^C 2D INADEQUATE from a last-resort method to a strategically valuable tool.

However, these advances remain unevenly distributed. High-temperature 10 mm cryoprobes and DNP hardware are available at only a limited number of facilities worldwide. The 5.5-fold sensitivity gain of the 10 mm cryoprobe and the ~2-fold gain of INADEQUATE CR are substantial, yet they do not fully overcome the natural-abundance penalty. NUS, now routine on modern spectrometers, can reduce effective NMR time by 50–75% but has not yet been applied to liquid 2D INADEQUATE. Integration of NUS with cryoprobe or DNP enhancement represents an obvious but currently unexploited opportunity.

Looking forward, the convergence of higher-field magnets, improved cryoprobes, AI-assisted denoising and automated analysis, NUS, and integration with computational chemistry will continue to lower practical barriers. For favorable cases involving ^13^C-enriched samples, small molecules, or cryoprobe-equipped spectrometers, NMR time has already been reduced to less than an hour. As these technologies further mature, 2D INADEQUATE is expected to transition from a specialist technique to a more routinely accessible tool in the structural-chemistry workflow.

## Figures and Tables

**Table 1:** Comparison of existing liquid-state INADEQUATE reviews.

Feature	Buddrus & Lambert (2002) [6]	Uhrín (2010) [7]	Present Review
Scope	Methodology and applications; includes solid-state INADEQUATE	Methodology-focused; liquid-state emphasis	Applications-focused; liquid-state only
Nuclei covered	^13^C–^13^C, ^29^Si–^29^Si, ^183^W–^183^W	^13^C–^13^C, ^29^Si–^29^Si, ^15^N–^15^N, ^77^Se–^77^Se	^13^C–^13^C exclusively
Pulse-sequence detail	Moderate	Extensive	Concise
Application coverage	Limited number of illustrative examples	Few application examples (up to 2009)	100+ application entries
Methodological developments after 2010	Not covered	Not covered	1.5 mm cryoprobe DNP-INADEQUATE, computerized analysis (FRED, INETA, SATIS), AI/DL denoising
Experimental parameters (mass, field, time)	Not tabulated	Not tabulated	Tabulated (Table 3)
Practical guidance and troubleshooting	Minimal	Minimal	Maximal (cf. Section 7)
Limitations discussion	Brief	Brief	Expanded: sensitivity, NMR time, strong coupling, quaternary-carbon invisibility in INEPT variants
Unique contribution	First comprehensive review of INADEQUATE methodology	Detailed pulse-sequence theory	Most comprehensive application compendium to date; bridges methodology and real-world structural elucidation

**Table 2:** Quantitative comparison of methodological improvements to liquid-state 2D INADEQUATE.

Methodological Developments	Method/Variant	Sensitivity Gain/Performance and NMR Time Impact	Key Limitation	Ref.
**Pulse-sequence and coherence-transfer variants**	J-INADEQUATE	Minimizing resonance-offset effects J-compensation, higher S/N than that of conventional INADEQUATE	Extra pulses and delays cause RF imperfection and relaxation losses	[118]
	INADEQUATE CR	2-fold sensitivity enhancement, reducing NMR time by approximately 4-fold	Requires complex phase-cycling; vulnerable to RF inhomogeneity, J-mismatch, and relaxation losses during long delays	[1,122,123,145,146,147]
	Adiabatic-pulse variant	No explicit sensitivity factor; tolerates 200 ppm offsets	Long chirp pulses, relaxation losses	[128]
	STRONG INADEQUATE	Detecting small C-C coupling constants	T_1_ noise, approximate J required, relaxation losses	[130]
	Artifact-free phase cycling	Cross-peak enhancement. Enables faster repetition without artifact accumulation	DQ artifacts persist for long-T_1_ carbons; needs 64-step cycling, careful calibration; not universally artifact-free	[126]
	DNP-INADEQUATE (Overhauser, steady-state)	Provides continuous, steady-state hyperpolarization that enables repeated signal averaging without the signal decaying over time	Primarily to small molecules and makes it largely unsuitable for macromolecules; specialized DNP hardware	[131]
**Polarization-transfer variants**	DEPT-INADEQUATE	≥2-fold S/N enhancement (2.1 × observed)	Requires extensive phase-cycling; highly sensitive to hardware misadjustments, where minor pulse errors cause >50% signal loss, quaternary-quaternary pairs invisible	[117]
	INEPT-INADEQUATE (^13^C-detected)	~2.6 × S/N, ~6 × time saving	Quaternary-quaternary pairs invisible	[116,127]
	INEPT-INADEQUATE (^1^H-detected)	~8-fold S/N improvement over ^13^C-detected INADEQUATE	Poor ^1^H resolution for polyolefins (~1 ppm range); quaternary pairs invisible	[124]
**Probe hardware**	5 mm ^13^C Cryoprobe	Up to ~4 × S/N over room-temperature probes	Sample volume limited to 5 mm tubes	[44,84]
	High-temperature 10 mm ^13^C Cryoprobe	5.5 × over conventional 10 mm broadband probe at 125 °C Reduced NMR time from ~2.6 months to 2.6 days	Limited availability; requires thermally stable samples	[105,106]
	1.5 mm ^13^C cryogenic nanoprobe	Major reduction in sample-mass requirement	Very limited sample volume	[47,129]
**Data processing and automated analysis**	Symmetry-based processing (S1/S2; SATIS)	2 × S/N (S1/S2), equivalent to 4 × NMR time reduction Post-processing only; no additional spectrometer time	Weak signals buried in noise remain difficult; degenerate shifts unresolved	[125,148]
	Automated analysis (CCBond; FRED)	Reliable bond detection at RMS S/N as low as 2.5 Reduces interpretation time from days to hours	Near-degenerate shifts and severe second-order effects remain problematic	[119,120,121,149,150,151,152]
	Network analysis (INETA; PyINETA)	Identifying metabolites in complex mixtures without chromatographic separation	Requires ^13^C labeling	[115,129]

**Table 3:** Representative Experimental Parameters for Selected 2D INADEQUATE Applications.

Compound/System	Sample Mass/Concentration	Field ^a^ (MHz)	Probe Type	NMR time	^13^C Enrichment	Ref.
**Brevetoxin B (BTX-B)**	1.5 mg/3.2mM (C_6_D_6_)	500	NR	NR	^l3^CH_3_^13^CO_2_Na labeled BTX-B	[12]
**2,3:5,6-di-O-isopropylidene-** **α** **-D-mannofuranose**	10 mg in CDCl_3_	500	^13^C optimized cryoprobe	9 h	Nat. ab.	[44]
**Karlotoxin-2**	1.3 mg	600	Dual cryoprobe	NR	10% ^13^C	[46]
**Histidine ^b^**	1.1 mg in 35 μL D_2_O/~200 mM	600	1.5 mm ^13^C HTS cryoprobe	48 h	Nat. ab.	[47]
**Vialisyl A**	NR	600	5 mm ^13^C observe cryoprobe	~90 h	Nat. ab.	[49]
**Pyrenacantha heterodimer**	100 μmol (DMSO-d_6_/acetone-d_6_ (80/20, *v*/*v*))	950	Standard broadband	79 h	Nat. ab.	[50]
**Dehydrocrebanine**	10.4 mg in 250 μL CDCl_3_	800	cryoprobe	55 h	Nat. ab.	[58]
**7,7a,13,14-tetrahydro-6H-cyclobuta[b]pyrimido [1,2-a:3,4-a′]diindole**	70 mg in 0.7 mL CDCl_3_	600	^13^C optimized 5 mm DCH cryoprobe	66 h	Nat. ab.	[84]
**Polypropylene (methine region)**	40 mg in 1 mL TCE-d_2_	600	5 mm	9 d	Nat. ab.	[103]
**Isotactic polypropylene 2,1-regioerrors ^c^**	1–2 g in 1–2 g stock solvent (1,4-PDCB-d_4_/ODCB)	400	10 mm HT ^13^C cryoprobe	2–3 d	Nat. ab.	[105]
**Poly(propylene-co-1-octene)**	1.5 g in 1.5 g stock solvent (1,4-PDCB-d_4_/ODCB)	400	10 mm HT DUAL cryoprobe	2.6 d	Nat. ab.	[106]
**Propene (gas phase) ^d^**	0.51 M	500	5 mm ^13^C-observe cryoprobe	14 h	Nat. ab.	[112]
**Methyl salicylate ^e^**	80%/20% (*v*/*v*) (CDCl_3_)	NR	^13^C optimized autoswitchable probe	3 h	Nat. ab.	[127]
**Histidine ^f^**	2 mM (labeled)/200 mM (Nat. ab.)	600	1.5 mm ^13^C HTS cryoprobe	4 h (labeled) vs. 48 h (nat. ab.)	99% ^13^C vs. Nat. ab.	[129]
**p-Cymene ^g^**	~17 mg	400	custom-modified liquid-state DNP probe	140 h	Nat. ab.	[131]
**3-Hydroxypregnane-2,16-dione**	11 mg/30 μmol (CDCl_3_)	500	Nanoprobe	62 h	Nat. ab.	[152]

Footnotes: NR = not reported in the original publication; Nat. ab. = natural abundance; HT = high-temperature. ^a 1^H Larmor frequency. ^b^ Adiabatic pulses; ~2 weeks would be required with a 5 mm probe. ^c^ 5.5-fold sensitivity gain over a conventional 10 mm broadband probe at 125 °C. ^d^ 30 °C; 2 s recycle delay (T_1_ = 0.43–2.4 s). ^e^ O_2_ pressurized (3 atm), 15 °C. ^f^ Demonstrates the effect of isotopic enrichment on NMR time. ^g^ Steady-state Overhauser DNP, 263 GHz irradiation.

## Data Availability

No new data were created in this study.

## Abbreviations

The following abbreviations are used in this manuscript:

2D	Two-Dimensional
INADEQUATE	Incredible Natural Abundance Double Quantum Transfer Experiment
NMR	Nuclear Magnetic Resonance
C-C	Carbon–Carbon
C-H	Carbon–Proton
HSQC	Heteronuclear Single Quantum Coherence
HMBC	Heteronuclear Multiple Bond Correlation
J_CC_	^13^C–^13^C Homonuclear Coupling Constant
J_CH_	^1^H–^13^C Heteronuclear Coupling Constant
τ	Echo Delay Time
t_1_	Evolution Delay Time
AQ	FID Acquisition Time
DQ	Double-Quantum
SQ	Single-Quantum
ω	Angular Frequency
F_1_	Indirect Dimension
F_2_	Direct Dimension
S/N	Signal-to-Noise Ratio
cryoprobe	Cryogenic Probe
INEPT	Insensitive Nuclei Enhanced by Polarization Transfer
CR	Composite Refocusing
J-INADEQUATE	J-Compensated INADEQUATE
RF	Radio Frequency
B_1_	Radio Frequency Magnetic Field Strengths
PFGs	Pulsed Field Gradients
ORE	Off-Resonance Effects
DQC	Double-Quantum Coherence
SQC	Single-Quantum Coherence
AP-SQC	Antiphase Single-Quantum Coherence
MQC	Multiple Quantum Coherence
SYMONA	Symmetrical Nuclear Magnetic Analysis
DNP	Dynamic Nuclear Polarization
PHIP	Parahydrogen-Induced Hyperpolarization
DEPT	Distortionless Enhancement by Polarization Transfer
RINEPT	Refocused Insensitive Nuclei Enhanced by Polarization Transfer
AIOQ-RINEPT	AI-Optimized Quantitative RINEPT
PyINETA	Python-Based INADEQUATE Network Analysis
Cr(acac)_3_	Chromium(III) Acetylacetonate
T_1_	Longitudinal Relaxation Time
T_2_	Transverse Relaxation Time
DFT	Density Functional Theory
iPP	Isotactic Polypropylene
RE	Regioerror
H-T	Head-to-Tail
H-H	Head-to-Head
T-T	Tail-to-Tail
1-r	Single Inverted Unit
2-r	Double Inverted Units
2U-r	Consecutive Double Inverted Units
LR-HSQMBC	Long-Range Heteronuclear Single-Quantum Multiple-Bond Correlation
COSY	Correlation Spectroscopy
TOCSY	Total Correlation Spectroscopy
NOESY	Nuclear Overhauser Effect Spectroscopy
HOESY	Heteronuclear Overhauser Effect Spectroscopy
AI	Artificial Intelligence
DL	Deep Learning
